# Synthesis, Structural Determination, and Antifungal Activity of Novel Fluorinated Quinoline Analogs

**DOI:** 10.3390/molecules28083373

**Published:** 2023-04-11

**Authors:** Xinpeng Sun, Wei Yu, Lijing Min, Liang Han, Xuewen Hua, Jianjun Shi, Nabo Sun, Xinghai Liu

**Affiliations:** 1 College of Biology and Environmental Engineering, Zhejiang Shuren University, Hangzhou 310015, China; 2 College of Chemical Engineering, Zhejiang University of Technology, Hangzhou 310014, China; 3 Key Laboratory of Vector Biology and Pathogen Control of Zhejiang Province, College of Life Science, Huzhou University, Huzhou 313000, China; 4 College of Agriculture, Liaocheng University, Liaocheng 252000, China; 5 College of Chemistry and Chemical Engineering, Huangshan University, Huangshan 245041, China

**Keywords:** fluorinated quinoline, synthesis, antifungal activity

## Abstract

A series of new fluorinated quinoline analogs were synthesized using Tebufloquin as the lead compound, 2-fluoroaniline, ethyl 2-methylacetoacetate, and substituted benzoic acid as raw materials. Their structures were confirmed by ^1^H NMR, ^13^C NMR, and HRMS. The compound 8-fluoro-2,3-dimethylquinolin-4-yl 4-(*tert*-butyl)benzoate (**2b**) was further determined by X-ray single-crystal diffraction. The antifungal activity was tested at 50 μg/mL, and the bioassay results showed that these quinoline derivatives had good antifungal activity. Among them, compounds **2b**, **2e**, **2f**, **2k**, and **2n** exhibited good activity (>80%) against *S. sclerotiorum*, and compound **2g** displayed good activity (80.8%) against *R. solani*.

## 1. Introduction

Nitrogen-containing heterocycles are important skeletons in synthetic chemistry [1,2,3,4,5]. In the development of heterocyclic pesticides, quinoline compounds, as a class of heterocyclic compounds with a quinoline skeleton [6], can be easily modified in structure, and play an important role in the development of new pesticides. Quinoline compounds not only have good antifungal [7,8,9,10], herbicidal [11], and anti-insecticidal [12] activities, but also have anticancer [13,14], antimalarial [15,16,17], and antituberculosis [18,19] activities. Since Runge [20] first extracted quinoline from coal tar, many natural quinoline compounds were discovered. 

**Figure 1:**
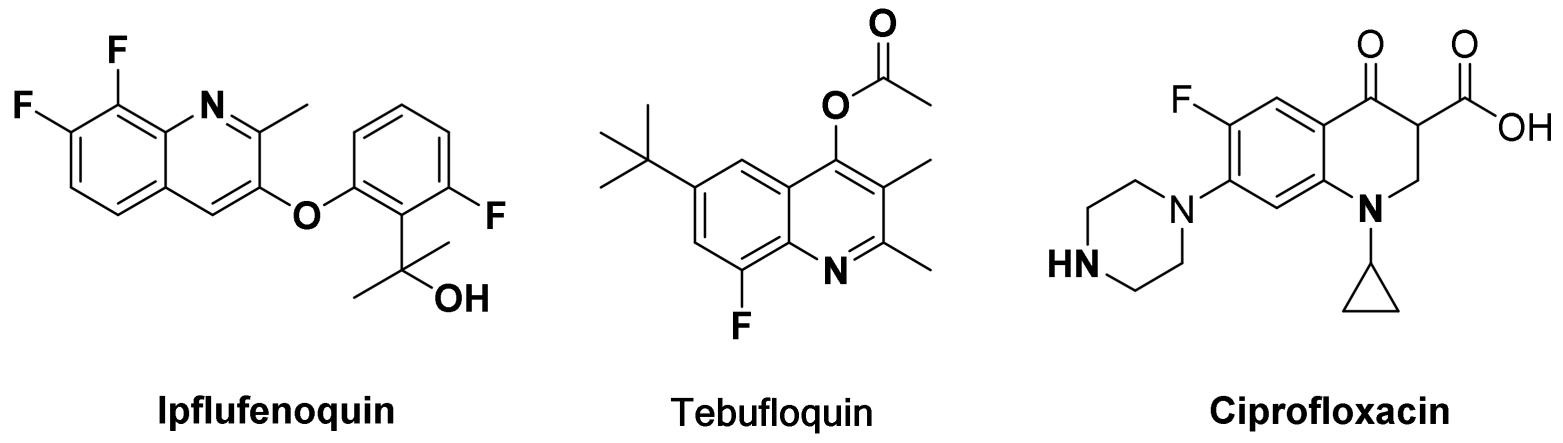
Some commercialized pesticides or drugs with fluorinated quinoline derivatives.

Among the more than 1200 commercialized pesticides, 424 pesticides contain at least one fluorine atom [21]. After organic compounds are fluorinated, their physical and chemical properties such as lipophilicity, water solubility, and metabolic stability will undergo significant changes [22], due to that they [23,24] have the characteristics of small radius and large electronegativity. Therefore, many commercial medicines or pesticides heterocycles containing fluorine atoms were commercialized. For example (Figure 1), the fluoroquinoline fungicide Ipflufenoquin [25] was recently developed by Japan’s Soda Company. The Meiji Fruit Industry in Japan has developed the fungicide Tebufloquin [26], which has excellent control effects on rice blast, and the commercial antifungal Ciprofloxacin.

**Figure 2:**
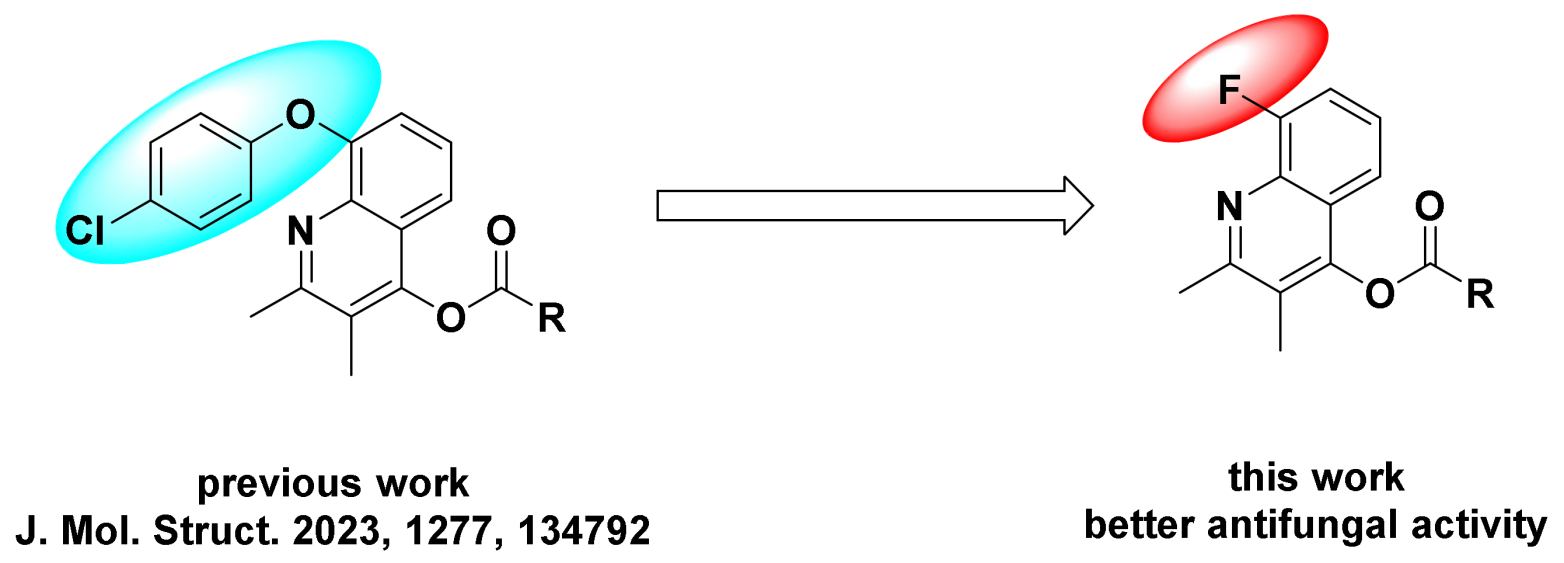
Design strategy of the title compounds **2a**–**2p** [32].

In our previous work [27,28,29,30,31], many compounds based on the natural quinoline structure were synthesized and showed good activity. In recent work [32], we found that in the 8-position quinoline ring added *p*-chlorophenoxy group, some compounds showed certain activity. As we continued our effort to develop high-efficiency and low-toxicity new quinoline-based fungicides, the natural quinoline structure was selected as key core, 8-position of quinoline ring was replaced by fluorine atom, and the 4-position of hydroxyl on the quinoline ring was esterified (Figure 2 [32]). All new quinoline compounds were characterized by ^1^H NMR, ^13^C NMR, and HRMS, and their antifungal activity was tested.

## 2. Results and Discussion

### 2.1. Synthesis and Spectra Analysis

**Scheme 1:**
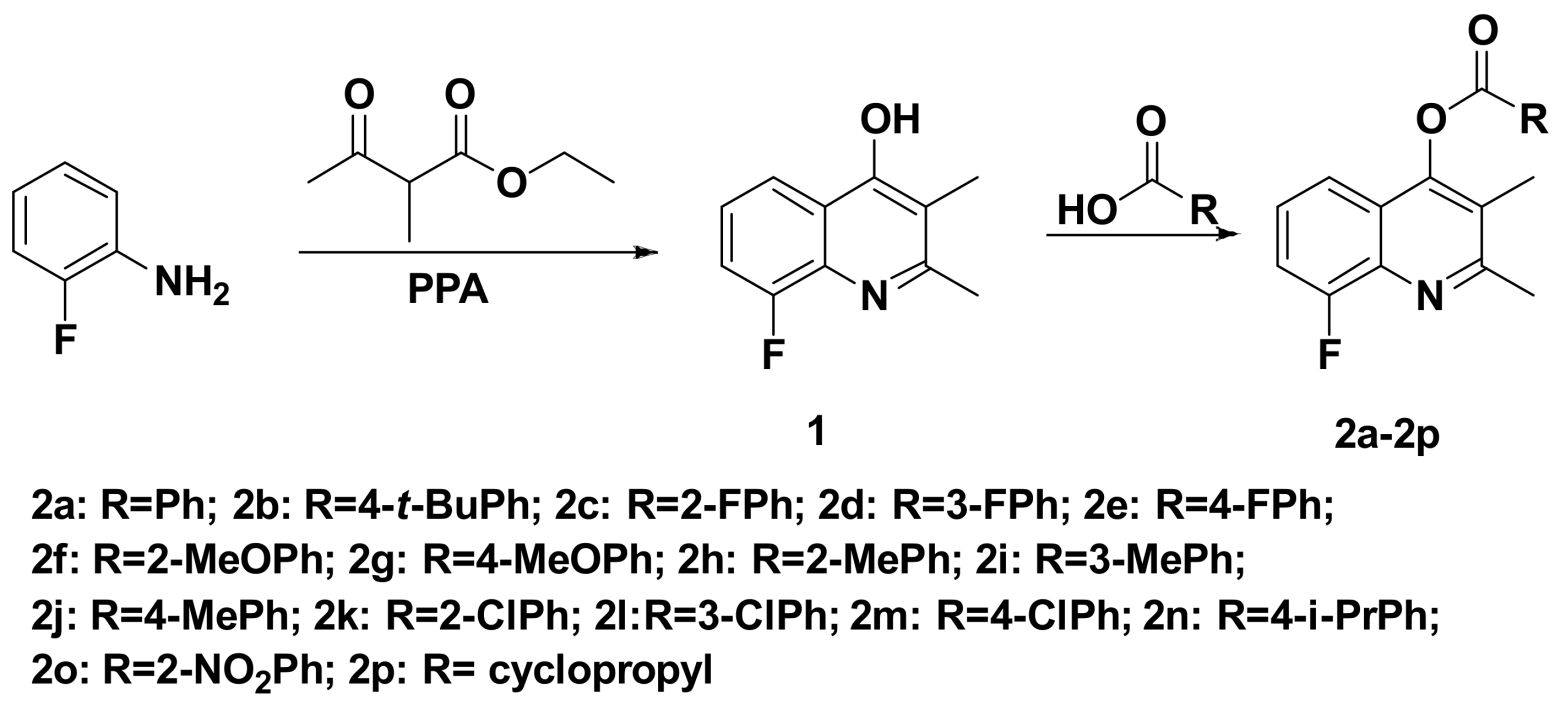
Synthesis route of title compounds **2a**–**2p**.

The synthetic route of title fluorinated quinoline analogs is shown in Scheme 1. First, 2-fluorophenol was directly reacted with ethyl 2-methylacetoacetate under polyphosphoric acid (PPA) to give 2,3-dimethyl-4-hydroxyquinoline intermediate **1**. The PPA was used as both a solvent and an acidic catalyst, and the reactants are directly one-step synthesis of quinoline rings. Then, intermediate **1** undergoes esterification with various substituted benzoic acids to generate the target compound. When EDC•HCl and DMAP are used as condensation agent, the solvent has a great influence on the yield of this reaction. It was found that using DMF as solvent can give higher yield than using dichloromethane (DCM) as solvent (Table 1).

The two CH_3_ protons are at 2.3 and 2.8 ppm, respectively. The protons of the benzene ring are assigned at 7~9 ppm. Taking compound **2b** as an example, the protons of two methyl groups can be found at *δ* 2.32 ppm and *δ* 2.80 ppm as singlets in the ^1^H NMR, while in the ^13^C NMR data, they are found at 12.87 ppm and 24.37 ppm, respectively. The C=O was found at 163.73 ppm in the ^13^C NMR.

### 2.2. Structure Determination 

A colorless crystal of 8-fluoro-2,3-dimethylquinolin-4-yl 4-(*tert*-butyl)benzoate, **2b**, suitable for X-ray diffraction study, was cultivated in the test tube from EtOH by self-volatilization. A crystal with dimensions of 0.36 mm × 0.28 mm × 0.18 mm was mounted on a Bruker APEX-II CCD diffractometer equipped with graphite-monochromatic Mo*Kα* radiation (*λ* = 0.71073 Å). The crystal structure was solved by direct methods with SHELXS-97 [33] and refined by full-matrix least-squares refinements based on *F*^2^ with SHELXL-97. All nonhydrogen atoms were refined anisotropically, and all hydrogen atoms were located in the calculated positions and refined with a riding model. The detailed crystal data are listed in Table 2.

### 2.3. Fungicidal Activity and SAR

The antifungal activities of compounds **2a**–**2p** against ten phytopathogenic fungi are listed in Table 3. Generally, these compounds showed good antifungal activity. For example, compounds **2b**(4-Bu), **2e**(4-F), **2g**(4-OMe) and **2p** displayed moderate activity (40~60%) against *A. solani*. For the *P. oryae*, compounds **2b**, **2d**, **2f,** and **2p** also exhibited moderate activity (40~60%), which is the same as the *A. solani*. The inhibition rate of compound **2f** against *P. capsicum* reached 58.1%, which was the same as that of the positive control Tebufloquin (58.1%). Compounds **2e** and **2j** were both 45% inhibitory to *F. oxysporum*, which is a little better than the positive control Tebufloquin (42.9%). Among these fungi, compounds **2b**, **2e**, **2f**, **2k**, and **2n** possessed good activity (>80%) against *S. sclerotiorum*, which were higher than the positive control Tebufloquin (75.0%). Compounds **2b** and **2d** also had good activity (53.8%) against *B. cinerea*, which was a little lower than that of the positive control Tebufloquin (56.7%), but the activity of compound **2n** (57.7%) was a little higher than that of the positive control Tebufloquin (56.7%). For the *R. solani*, compounds **2g** (80.8%) and **2p** (76.9%) exhibited good activity, which were higher than the positive control Tebufloquin (69.7%). The inhibition rates of compounds **2b**, **2f**, and **2n** against *C. arachidicola* were 46.7%, 46.7%, and 60%, respectively, which were higher than the positive control Tebufloquin (37.5%). The inhibitory rates of compounds **2e**, **2f**, **2k**, and **2n**, against *P. piricola* were 72.0%, 76.0%, 76.0%, and 76.0%, respectively, which were also higher than the positive control Tebufloquin (65.4%). Notably, all the title compounds had lower inhibition rates against *G. zeae*; only compounds **2b** and **2n** exceeded 30%. From Table 3, it can be concluded that substitution on the benzene ring can influence the activity. The 4-position of the benzene ring had an electron-given group with better activity, such as compounds **2b**(4-Bu), **2g**(4-MeO), **2j**(4-Me), and **2n**(4-i-Pr), while the 4-position of the benzene ring had an electron-withdraw group, which had low activity, except for compound **2e**(4-F), which may be the influence of the fluorine atom. When the R was an alkyl group, such as compound **2p** (cyclopropyl group), it also exhibited good activity.

## 3. Materials and Methods

### 3.1. Instruments 

Melting points were determined using an X-4 apparatus and uncorrected. ^1^H NMR and ^13^C NMR spectra were measured on a Bruker AC-P500 and AC-P400 instrument using TMS as an internal standard and deuterated chloroform, CDCl_3_, as the solvent. HR-ESI-MS was tested using an Agilent 1100 HPLC-JEOL AccuTOF instrument. All reagents were of analytical grade or were freshly prepared before use.

### 3.2. Synthesis 

#### 3.2.1. Synthesis of Intermediate **1**

The synthetic route is shown in Scheme **1**. In a 250 mL three-necked flask, 2-fluoroaniline (11.11 g, 100.00 mmol), ethyl 2-methylacetoacetate (14.42 g, 100.00 mmol), and polyphosphoric acid (50.69 g, 150.00 mmol) were added, and the mixture was heated at 150 °C. After the reaction was completed, the mixture was cooled to room temperature. The three-necked flask was placed in an ice bath, and the pH was adjusted to 7–8 by 10% aqueous sodium hydroxide solution. Then, it was filtered and dried to give 8-fluoro-2,3-dimethylquinolin-4-ol (intermediate **1**), white solid with the yield of 89.2%, mp 230–231 °C. ^1^H NMR (500 MHz, DMSO-*d*_6_) *δ*: 11.36 (s, 1H, OH), 7.88 (d, *J* = 8.1 Hz, 1H, Ph), 7.52–7.46 (m, 1H, Ph), 7.26–7.19 (m, 1H, Ph), 2.43 (s, 3H, CH_3_), 1.98 (s, 3H, CH_3_). 

#### 3.2.2. Synthesis of Target Compounds **2**

The synthetic route is shown in Scheme 1. In a 50 mL round-bottomed flask, intermediate **1** (0.20 g, 1.05 mmol), substituted benzoic acid (1.16 mmol), EDC•HCl (0.24 g, 1.26 mmol), 4-dimethylaminopyridine (DMAP) (0.13 g, 1.05 mmol), and DMF (10 mL) were stirred at room temperature. After the reaction was completed, water (30 mL) was added, it was extracted with ethyl acetate (10 mL × 3), and the organic phases were combined, washed with saturated brine (10 mL × 3), dried over anhydrous sodium sulfate, filtered, and purified by column chromatography to obtain the target compounds **2a**–**2p**.

*8-fluoro-2,3-dimethylquinolin-4-yl benzoate* (**2a**). White solid, yield 56.1%, mp 176~178 °C; ^1^H NMR (500 MHz, CDCl_3_) *δ*: 8.36–8.30 (m, 2H, Ph), 7.80–7.69 (m, 1H, Ph), 7.60 (t, *J* = 7.8 Hz, 2H, Ph), 7.56–7.49 (m, 1H, Ph), 7.42–7.29 (m, 2H, Ph), 2.81 (s, 3H, CH_3_), 2.33 (s, 3H, CH_3_); ^13^C NMR (151 MHz, CDCl_3_) *δ*: 163.75, 160.83, 157.74 (d, *J* = 255.8 Hz), 151.72 (d, *J* = 4.5 Hz), 137.55 (d, *J* = 12.2 Hz), 134.38, 130.49 (2C), 128.97 (2C), 128.21, 125.99 (d, *J* = 8.2 Hz), 123.58 (d, *J* = 2.4 Hz), 123.23, 116.72 (d, *J* = 4.7 Hz), 113.24 (d, *J* = 19.2 Hz), 24.39, 12.89; HRMS (ESI) for C_18_H_14_FNO_2_ *m*/*z*: Calculated, 296.1081, found, 296.1087 [M + H]^+^.

*8-fluoro-2,3-dimethylquinolin-4-yl 4-(tert-butyl)benzoate* (**2b**). White solid, yield 59.3%, mp 171~173 °C; ^1^H NMR (500 MHz, CDCl_3_) *δ*: 8.29–8.19 (m, 2H, Ph), 7.61 (d, *J* = 8.6 Hz, 2H, Ph), 7.56–7.50 (m, 1H, Ph), 7.40–7.30 (m, 2H, Ph), 2.80 (s, 3H, CH_3_), 2.32 (s, 3H, CH_3_), 1.41 (s, 9H, C(CH_3_)_3_); ^13^C NMR (151 MHz, CDCl_3_) *δ*: 163.73, 160.82, 158.37, 157. 73 (d, *J* = 256.7 Hz), 151.82 (d, *J* = 4.5 Hz), 137.52 (d, *J* = 12.1 Hz), 130.44 (2C), 125.97 (2C), 125.91 (d, *J* = 8.2 Hz), 125.38, 123.68 (d, *J* = 2.3 Hz), 123.28, 116.79 (d, *J* = 4.7 Hz), 113.18 (d, *J* = 19.2 Hz), 35.35, 31.11 (3C), 24.37, 12.87; HRMS (ESI) for C_22_H_22_FNO_2_ *m*/*z*: Calculated, 352.1707, found, 352.1713 [M + H]^+^.

*8-fluoro-2,3-dimethylquinolin-4-yl 2-fluorobenzoate* (**2c**). White solid, yield 67.2%, mp 144~146 °C; ^1^H NMR (500 MHz, CDCl_3_) *δ*: 8.24–8.17 (m, 1H, Ph), 7.75–7.67 (m, 1H, Ph), 7.58 (d, *J* = 8.1 Hz, 1H, Ph), 7.44–7.28 (m, 4H, Ph), 2.81 (s, 3H, CH_3_), 2.35 (s, 3H, CH_3_); ^13^C NMR (151 MHz, CDCl_3_) *δ*: 162.54 (d, *J* = 262.0 Hz), 161.43 (d, *J* = 4.0 Hz), 160.87, 157.70 (d, *J* = 255.9 Hz), 151.46 (d, *J* = 4.6 Hz), 137.50 (d, *J* = 12.2 Hz), 136.04 (d, *J* = 9.1 Hz), 132.80, 126.11 (d, *J* = 8.2 Hz), 124.55 (d, *J* = 3.9 Hz), 123.39 (d, *J* = 2.4 Hz), 123.18, 117.53 (d, *J* = 22.1 Hz), 116.89 (d, *J* = 9.7 Hz), 116.75 (d, *J* = 4.7 Hz), 113.32 (d, *J* = 19.2 Hz), 24.32, 12.96; HRMS (ESI) for C_18_H_13_F_2_NO_2_ *m*/*z*: Calculated, 314.0987, found, 314.0993 [M + H]^+^.

*8-fluoro-2,3-dimethylquinolin-4-yl 3-fluorobenzoate* (**2d**). White solid, yield 66.2%, mp 146~148 °C; ^1^H NMR (500 MHz, CDCl_3_) *δ*: 8.17–8.09 (m, 1H, Ph), 8.03–7.97 (m, 1H, Ph), 7.63–7.56 (m, 1H, Ph), 7.54–7.49 (m, 1H, Ph), 7.48–7.42 (m, 1H, Ph), 7.42–7.33 (m, 2H, Ph), 2.81 (s, 3H, CH_3_), 2.32 (s, 3H, CH_3_); ^13^C NMR (151 MHz, CDCl_3_) *δ*: 162.80 (d, *J* = 249.2 Hz), 162.65 (d, *J* = 3.1 Hz), 160.88, 157.75 (d, *J* = 256.1 Hz), 151.48 (d, *J* = 4.4 Hz), 137.53 (d, *J* = 12.1 Hz), 130.73 (d, *J* = 7.8 Hz), 130.29 (d, *J* = 7.5 Hz), 126.25 (d, *J* = 3.1 Hz), 126.15 (d, *J* = 8.2 Hz), 123.36 (d, *J* = 2.3 Hz), 123.16, 121.56 (d, *J* = 21.3 Hz), 117.35 (d, *J* = 23.3 Hz), 116.54 (d, *J* = 4.7 Hz), 113.37 (d, *J* = 19.2 Hz), 24.34, 12.90; HRMS (ESI) for C_18_H_13_F_2_NO_2_ *m*/*z*: Calculated, 314.0987, found, 314.0993 [M + H]^+^.

*8-fluoro-2,3-dimethylquinolin-4-yl 4-fluorobenzoate* (**2e**). White solid, yield 48.5%, mp 152~154 °C; ^1^H NMR (500 MHz, CDCl_3_) *δ*: 8.48–8.22 (m, 2H, Ph), 7.55–7.46 (m, 1H, Ph), 7.41–7.32 (m, 2H, Ph), 7.31–7.25 (m, 2H, Ph), 2.81 (s, 3H, CH_3_), 2.32 (s, 3H, CH_3_); ^13^C NMR (151 MHz, CDCl_3_) *δ*: 166.65 (d, *J* = 256.6 Hz), 162.78, 160.88, 157.73 (d, *J* = 256.0 Hz), 151.60 (d, *J* = 4.5 Hz), 137.50 (d, *J* = 12.2 Hz), 133.19 (d, *J* = 9.6 Hz, 2C), 126.09 (d, *J* = 8.2 Hz), 124.43 (d, *J* = 3.0 Hz), 123.50 (d, *J* = 2.5 Hz), 123.24, 116.60 (d, *J* = 4.7 Hz), 116.29 (d, *J* = 22.2 Hz, 2C), 113.33 (d, *J* = 19.2 Hz), 24.31, 12.89; HRMS (ESI) for C_18_H_13_F_2_NO_2_ *m*/*z*: Calculated, 314.0987, found, 314.0993 [M + H]^+^.

*8-fluoro-2,3-dimethylquinolin-4-yl 2-methoxybenzoate* (**2f**). White solid; yield 51.3%; mp 129~130 °C; ^1^H NMR (500 MHz, CDCl_3_) *δ*: 8.15 (dd, *J* = 7.9, 1.6 Hz, 1H, Ph), 7.69–7.61 (m, 2H, Ph), 7.44–7.30 (m, 2H, Ph), 7.17–7.09 (m, 2H, Ph), 3.99 (s, 3H, OCH_3_), 2.80 (s, 3H, CH_3_), 2.36 (s, 3H, CH_3_); ^13^C NMR (151 MHz, CDCl_3_) *δ*: 163.20, 160.81, 160.16, 157.70 (d, *J* = 255.5 Hz), 151.88 (d, *J* = 4.5 Hz), 137.54 (d, *J* = 12.1 Hz), 135.08, 132.52, 125.85 (d, *J* = 8.2 Hz), 123.76 (d, *J* = 2.5 Hz), 123.29, 120.50, 117.93, 117.10 (d, *J* = 4.6 Hz), 113.10 (d, *J* = 19.2 Hz), 112.36, 56.04, 24.40, 12.94; HRMS (ESI) for C_19_H_16_FNO_3_ *m*/*z*: Calculated, 326.1187, found, 326.1192 [M + H]^+^.

*8-fluoro-2,3-dimethylquinolin-4-yl 4-methoxybenzoate* (**2g**). White solid, yield 57.3%, mp 86~88 °C; ^1^H NMR (500 MHz, CDCl_3_) *δ*: 8.35–8.20 (m, 2H, Ph), 7.55–7.50 (m, 1H, Ph), 7.40–7.30 (m, 2H, Ph), 7.09–7.03 (m, 2H, Ph), 3.94 (s, 3H, OCH_3_), 2.80 (s, 3H, CH_3_), 2.32 (s, 3H, CH_3_); ^13^C NMR (151 MHz, CDCl_3_) *δ*: 163.48, 162.31, 160.83, 157.73 (d, *J* = 255.7 Hz), 151.87 (d, *J* = 4.6 Hz), 137.52 (d, *J* = 12.0 Hz), 132.84 (2C), 125.89 (d, *J* = 8.2 Hz), 123.78 (d, *J* = 2.4 Hz), 123.32, 120.40, 116.82 (d, *J* = 4.7 Hz), 114.15 (2C), 113.17 (d, *J* = 19.2 Hz), 55.60, 24.38, 12.89; HRMS (ESI) for C_19_H_16_FNO_3_ *m*/*z*: Calculated, 326.1187, found, 326.1192 [M + H]^+^.

*8-fluoro-2,3-dimethylquinolin-4-yl 2-methylbenzoate* (**2h**). White solid, yield 60.5%, mp 150~152 °C; ^1^H NMR (500 MHz, CDCl_3_) *δ*: 8.39 (d, *J* = 7.8 Hz, 1H, Ph), 7.64–7.50 (m, 2H, Ph), 7.48–7.31 (m, 4H, Ph), 2.81 (s, 3H, CH_3_), 2.70 (s, 3H, CH_3_), 2.34 (s, 3H, CH_3_); ^13^C NMR (151 MHz, CDCl_3_) *δ*: 164.03, 160.83, 157.76 (d, *J* = 255.8 Hz), 151.80 (d, *J* = 4.5 Hz), 142.34, 137.55 (d, *J* = 12.1 Hz), 133.58, 132.41, 131.47, 126.99, 126.30, 125.98 (d, *J* = 8.2 Hz), 123.72 (d, *J* = 2.4 Hz), 123.25, 116.76 (d, *J* = 4.7 Hz), 113.21 (d, *J* = 19.2 Hz), 24.37, 22.10, 12.95; HRMS (ESI) for C_19_H_16_FNO_2_ *m*/*z*: Calculated, 310.1238, found, 310.1243 [M + H]^+^.

*8-fluoro-2,3-dimethylquinolin-4-yl 3-methylbenzoate* (**2i**). White solid, yield 63.1%, mp 126~128 °C; ^1^H NMR (500 MHz, CDCl_3_) *δ*: 8.17–8.07 (m, 2H, Ph), 7.58–7.51 (m, 2H, Ph), 7.48 (t, *J* = 7.9 Hz, 1H, Ph), 7.41–7.32 (m, 2H, Ph), 2.81 (s, 3H, CH_3_), 2.50 (s, 3H, CH_3_), 2.32 (s, 3H, CH_3_); ^13^C NMR (151 MHz, CDCl_3_) *δ*: 163.92, 160.85, 157.72 (d, *J* = 255.8 Hz), 151.80 (d, *J* = 4.5 Hz), 138.93, 137.51 (d, *J* = 12.2 Hz), 135.16, 130.97, 128.86, 128.12, 127.65, 125.97 (d, *J* = 8.2 Hz), 123.62 (d, *J* = 2.4 Hz), 123.26, 116.76 (d, *J* = 4.6 Hz), 113.24 (d, *J* = 19.2 Hz), 24.34, 21.34, 12.89; HRMS (ESI) for C_19_H_16_FNO_2_ *m*/*z*: Calculated, 310.1238, found, 310.1243 [M + H]^+^.

*8-fluoro-2,3-dimethylquinolin-4-yl 4-methylbenzoate* (**2j**). White solid, yield 66.5%, mp 122~124 °C; ^1^H NMR (500 MHz, CDCl_3_) *δ*: 8.21 (d, *J* = 8.2 Hz, 2H, Ph), 7.56–7.50 (m, 1H, Ph), 7.43–7.30 (m, 4H, Ph), 2.80 (s, 3H, CH_3_), 2.51 (s, 3H, CH_3_), 2.32 (s, 3H, CH_3_); ^13^C NMR (151 MHz, CDCl_3_) *δ*: 163.81, 160.84, 157.73 (d, *J* = 255.7 Hz), 151.82 (d, *J* = 4.5 Hz), 145.43, 137.51 (d, *J* = 12.2 Hz), 130.55 (2C), 129.68 (2C), 125.94 (d, *J* = 8.2 Hz), 125.43, 123.68 (d, *J* = 2.4 Hz), 123.29, 116.78 (d, *J* = 4.7 Hz), 113.21 (d, *J* = 19.2 Hz), 24.35, 21.87, 12.88; HRMS (ESI) for C_19_H_16_FNO_2_ *m*/*z*: Calculated, 310.1238, found, 310.1243 [M + H]^+^.

*8-fluoro-2,3-dimethylquinolin-4-yl 2-chlorobenzoate* (**2k**). White solid, yield 46.3%, mp 116~118 °C; ^1^H NMR (500 MHz, CDCl_3_) *δ*: 8.25 (dd, *J* = 7.8, 1.2 Hz, 1H, Ph), 7.66–7.56 (m, 3H, Ph), 7.51–7.47 (m, 1H, Ph), 7.44–7.39 (m, 1H, Ph), 7.39–7.34 (m, 1H, Ph), 2.82 (s, 3H, CH_3_), 2.37 (s, 3H, CH_3_); ^13^C NMR (151 MHz, CDCl_3_) *δ*: 162.43, 160.84, 157.71 (d, *J* = 255.7 Hz), 151.44 (d, *J* = 3.5 Hz), 137.53 (d, *J* = 11.9 Hz), 134.94, 134.03, 132.34, 131.81, 127.87, 127.07, 126.14 (d, *J* = 8.0 Hz), 123.37 (d, *J* = 2.6 Hz), 123.17, 116.74 (d, *J* = 4.0 Hz), 113.31 (d, *J* = 19.1 Hz), 24.35, 13.05; HRMS (ESI) for C_18_H_13_ClFNO_2_ *m*/*z*: Calculated, 330.0692, found, 330.0697 [M + H]^+^.

*8-fluoro-2,3-dimethylquinolin-4-yl 3-chlorobenzoate* (**2l**). White solid, yield 68.5%, mp 148~150 °C; ^1^H NMR (500 MHz, CDCl_3_) *δ*: 8.30 (t, *J* = 1.7 Hz, 1H, Ph), 8.24–8.16 (m, 1H, Ph), 7.74–7.68 (m, 1H, Ph), 7.57–7.48 (m, 2H, Ph), 7.42–7.34 (m, 2H, Ph), 2.81 (s, 3H, CH_3_), 2.32 (s, 3H, CH_3_); ^13^C NMR (151 MHz, CDCl_3_) *δ*: 162.59, 160.88, 157.74 (d, *J* = 256.1 Hz), 151.47 (d, *J* = 4.6 Hz), 137.51 (d, *J* = 12.2 Hz), 135.23, 134.43, 130.44, 130.32, 129.92, 128.59, 126.17 (d, *J* = 8.2 Hz), 123.34 (d, *J* = 2.4 Hz), 123.16, 116.53 (d, *J* = 4.7 Hz), 113.39 (d, *J* = 19.2 Hz), 24.32, 12.91; HRMS (ESI) for C_18_H_13_ClFNO_2_ *m*/*z*: Calculated, 330.0692, found, 330.0697 [M + H]^+^.

*8-fluoro-2,3-dimethylquinolin-4-yl 4-chlorobenzoate* (**2m**). White solid, yield 70.2%, mp 183~185 °C; ^1^H NMR (500 MHz, CDCl_3_) *δ*: 8.32–8.21 (m, 2H, Ph), 7.62–7.56 (m, 2H, Ph), 7.53–7.46 (m, 1H, Ph), 7.42–7.33 (m, 2H, Ph), 2.81 (s, 3H, CH_3_), 2.31 (s, 3H, CH_3_); ^13^C NMR (151 MHz, CDCl_3_) *δ*: 162.94, 160.85, 157.76 (d, *J* = 256.0 Hz), 151.50 (d, *J* = 4.6 Hz), 141.09, 137.55 (d, *J* = 12.2 Hz), 131.83 (2C), 129.40 (2C), 126.61, 126.10 (d, *J* = 8.2 Hz), 123.41 (d, *J* = 2.5 Hz), 123.17, 116.55 (d, *J* = 4.8 Hz), 113.33 (d, *J* = 19.2 Hz), 24.39, 12.90; HRMS (ESI) for C_18_H_13_ClFNO_2_ *m*/*z*: Calculated, 330.0692, found, 330.0697 [M + H]^+^.

*8-fluoro-2,3-dimethylquinolin-4-yl 4-isopropylbenzoate* (**2n**). White solid, yield 46.5%, mp 144~146 °C; ^1^H NMR (500 MHz, CDCl_3_) *δ*: 8.29–8.21 (m, 2H, Ph), 7.57–7.50 (m, 1H, Ph), 7.45 (d, *J* = 8.2 Hz, 2H, Ph), 7.40–7.30 (m, 2H, Ph), 3.13–2.99 (m, 1H, CH), 2.80 (s, 3H, CH_3_), 2.32 (s, 3H, CH_3_), 1.34 (s, 3H, CH_3_), 1.33 (s, 3H, CH_3_); ^13^C NMR (151 MHz, CDCl_3_) *δ*: 163.76, 160.82, 157.73 (d, *J* = 255.7 Hz), 156.14, 151.82 (d, *J* = 4.5 Hz), 137.53 (d, *J* = 12.2 Hz), 130.73 (2C), 127.11 (2C), 125.91 (d, *J* = 8.2 Hz), 125.76, 123.68 (d, *J* = 2.3 Hz), 123.28 (s), 116.80 (d, *J* = 4.7 Hz), 113.18 (d, *J* = 19.2 Hz), 34.47, 24.38, 23.69 (2C), 12.87; HRMS (ESI) for C_21_H_20_FNO_2_ *m*/*z*: Calculated, 338.1551, found, 338.1556 [M + H]^+^.

*8-fluoro-2,3-dimethylquinolin-4-yl 2-nitrobenzoate* (**2o**). White solid, yield 46.3%, mp 204~206 °C, ^1^H NMR (500 MHz, CDCl_3_) *δ*: 9.17 (t, *J* = 1.8 Hz, 1H, Ph), 8.64 (d, *J* = 7.7 Hz, 1H, Ph), 8.62–8.57 (m, 1H, Ph), 7.84 (t, *J* = 8.0 Hz, 1H, Ph), 7.51–7.46 (m, 1H, Ph), 7.43–7.35 (m, 2H, Ph), 2.83 (s, 3H, CH_3_), 2.34 (s, 3H, CH_3_); ^13^C NMR (151 MHz, CDCl_3_) *δ*: 161.77, 160.90, 157.78 (d, *J* = 256.3 Hz), 151.16 (d, *J* = 4.6 Hz), 148.64, 137.60 (d, *J* = 12.2 Hz), 135.99, 130.38, 129.98, 128.74, 126.32 (d, *J* = 8.3 Hz), 125.34, 123.08 (d, *J* = 2.4 Hz), 123.06, 116.32 (d, *J* = 4.7 Hz), 113.50 (d, *J* = 19.2 Hz), 24.41, 12.97; HRMS (ESI) for C_18_H_13_FN_2_O_4_ *m*/*z*: Calculated, 341.0932, found, 341.0938 [M + H]^+^.

*8-fluoro-2,3-dimethylquinolin-4-yl cyclopropanecarboxylate* (**2p**). White solid, yield 32.8%, mp 115~117 °C; ^1^H NMR (500 MHz, CDCl_3_) *δ*: 7.51 (d, *J* = 8.3 Hz, 1H, Ph), 7.44–7.38 (m, 1H, Ph), 7.37–7.30 (m, 1H, Ph), 2.77 (s, 3H, CH_3_), 2.27 (s, 3H, CH_3_), 2.09–2.02 (m, 1H, CH), 1.32–1.28 (m, 2H, CH_2_), 1.21–1.12 (m, 2H, CH_2_); ^13^C NMR (151 MHz, CDCl_3_) *δ*: 171.99, 160.75, 157.71 (d, *J* = 255.6 Hz), 151.42 (d, *J* = 4.5 Hz), 137.47 (d, *J* = 12.1 Hz), 125.88 (d, *J* = 8.2 Hz), 123.57 (d, *J* = 2.5 Hz), 123.06, 116.61 (d, *J* = 4.6 Hz), 113.13 (d, *J* = 19.2 Hz), 24.35, 12.71, 12.66, 9.57 (2C); HRMS (ESI) for C_15_H_14_FNO_2_ *m*/*z*: Calculated, 260.1081, found, 260.1087 [M + H]^+^.

### 3.3. Fungicide Bioassays

The fungicidal activities of title chiral niacinamide derivatives **2a**–**2p** were tested in vitro against A. solani (AS), G. zeae (GZ), P. oryae (PO), P. capsici (PC), S. sclerotiorum (SS), B. cinerea (BC), R. solani (RS), F. oxysporum (FO), C. arachidicola (CA), and P. piricola (PP), and their relative percent inhibition (%) was determined using the mycelium growth rate method according to the previous work [34,35]. Tebufloquin was used as the positive control. Each compound was dissolved in DMSO with 1% Tween to prepare the 500 μg/mL stock solution. The ten fungi were inoculated into a Petri dish containing 50 μg/mL stock solution and incubated in a 27 °C incubator. A DMSO solvent containing 1% Tween was used as a blank assay. The fungicidal effect was determined 48–72 h later. The inhibition rate of the compound compared to the blank assay was calculated by the following equation:

inhibition (%) = (CK − AI)/CK × 100%

where CK is the average diameter of the mycelium in the blank test, and AI is the average value of the mycelium in the presence of these compounds.

### 3.4. Crystal Structure

**Figure 3:**
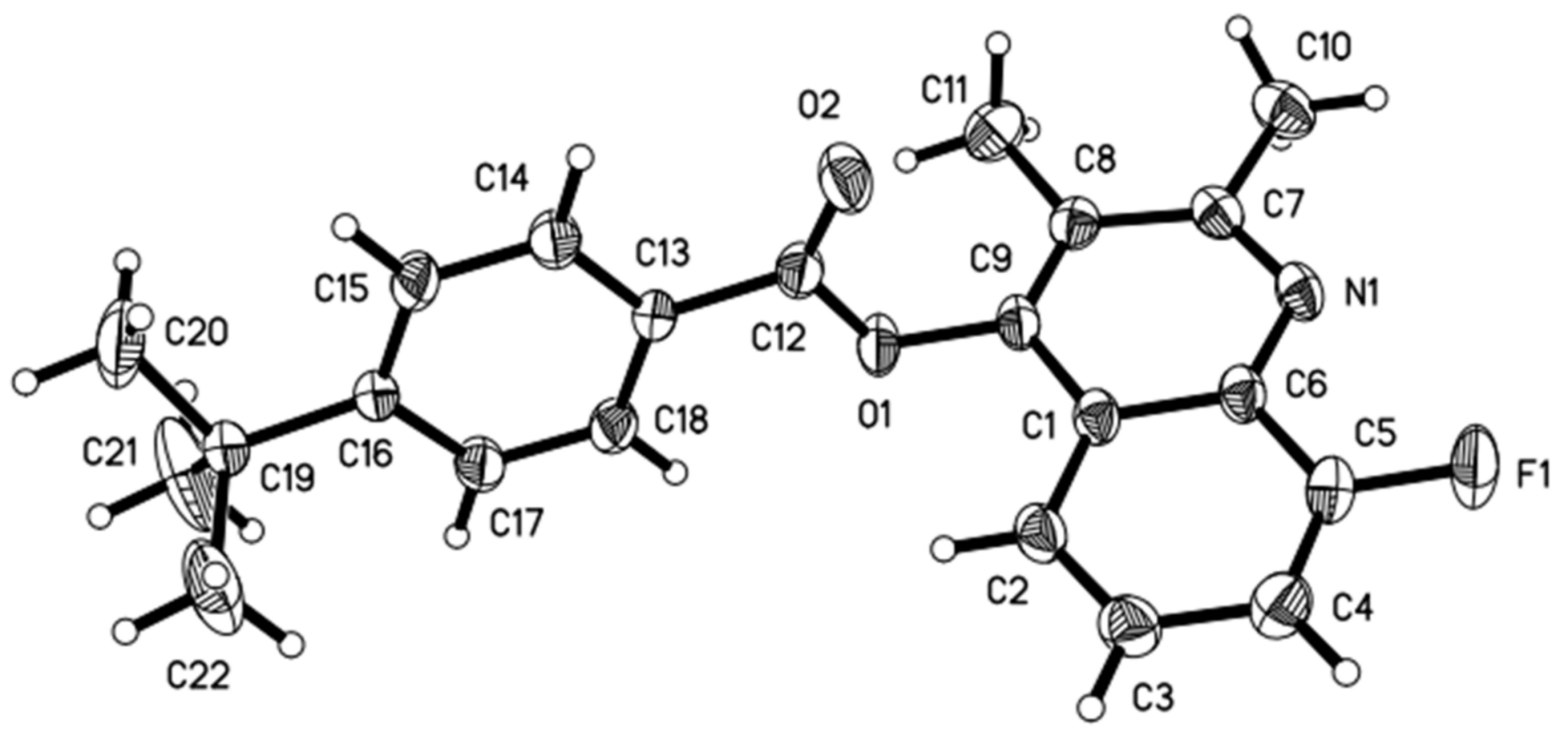
Molecular structure of the title compound **2b**.

**Figure 4:**
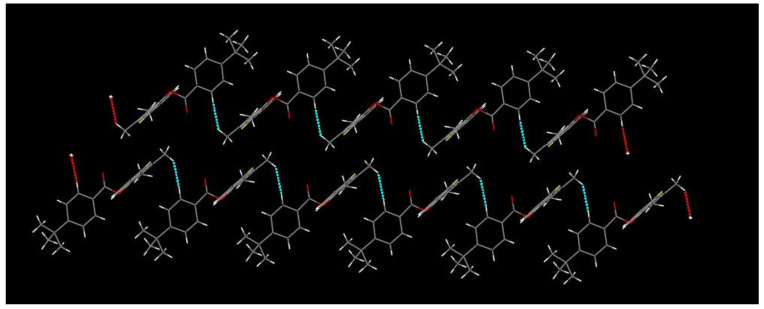
Chain structure of compound **2b**.

The structure of compound 8-fluoro-2,3-dimethylquinolin-4-yl 4-(*tert*-butyl)benzoate **2b** was further characterized by single-crystal X-ray diffraction, and is illustrated in Figure 3. As shown in Figure 3, the phenyl ring (C(13)~C(18) is nearly vertical with the quinoline ring, which has a dihedral angle (*θ*) of 76.2° with plane equation 2.499x + 3.956y + 7.124z = 5.9084 and 5.915x + 3.757y + −6.977z = 3.1104, respectively. The torsion angles of C(9)-O(1)-C(12)-C(13) and O(2)-C(12)-C(13)-C(14) were −171.3(3)° and −175.7(3)°, respectively, which showed that the ester group is nearly in the same plane as the phenyl ring. Compound **2b** had weak interaction, which formed an infinite one-dimensional chain structure along the a-axis (Figure 4).

## 4. Conclusions

In summary, sixteen new fluorinated quinoline compounds were synthesized, and their structures were characterized by ^1^H NMR, ^13^C NMR, X-ray diffraction, and HRMS. The antifungal activity results showed that some of the compounds exhibited good antifungal activity at 50 μg/mL. Among them, compounds **2b**, **2e**, **2f**, **2k**, and **2n** exhibited good antifungal activity (>80%) against *S. sclerotiorum*. The antifungal activity of compound **2g** against *R. solani* was 80.8%. This can give new clues for designing new highly active quinoline compounds in the next study.

## Figures and Tables

**Table 1:** Solvent effect of yield compound **2b**.

Solvent	Yield (%)
DMF	32.5
CH_2_Cl_2_	59.3

**Table 2:** Crystal data and structure refinement of compound **2b**.

Name	2b
Empirical formula	C_22_H_22_FNO_2_
Formula weight	351.40
Temperature/K	296(2)
Crystal system	triclinic
Space group	*P*-1
*a*/Å	8.4459(10)
*b*/Å	10.2380(12)
*c*/Å	11.7771(14)
*α*/°	101.100(6)
*β*/°	98.283(6)
*γ*/°	108.547(6)
Volume/Å^3^	924.05(19)
*Z*	2
*ρ*_calc_g/cm^3^	1.263
*μ*/mm^−1^	0.087
*F*(000)	372.0
Crystal size/mm^3^	0.36 × 0.28 × 0.18
Radiation	MoKα (*λ* = 0.71073)
2^Θ^ range for data collection/°	4.89 to 57.452
Index ranges	−11 ≤ h ≤ 10, −13 ≤ k ≤ 13, −15 ≤ l ≤ 15
Reflections collected	13,642
Independent reflections	4164 [*R*_int_ = 0.0755, *R*_sigma_ = 0.1961]
Data/restraints/parameters	4164/0/240
Goodness-of-fit on F^2^	1.172
Final *R* indexes [*I* ≥ 2*σ* (*I*)]	*R*_1_ = 0.1333, *wR*_2_ = 0.2614
Final *R* indexes [all data]	*R*_1_ = 0.3507, *wR*_2_ = 0.3608
Largest diff. peak/hole/e Å^−3^	1.19/−1.24

**Table 3:** The fungicidal activity (% inhibition) of compounds **2a**–**2p** and Tebufloquin (FP) at 50 μg/mL on *A. solani* (AS), *G. zeae* (GZ), *P. oryae* (PO), *P. capsici* (PC), *S. sclerotiorum* (SS), *B. cinerea* (BC), *R. solani* (RS), *F. oxysporum* (FO), *C. arachidicola* (CA), and *P. piricola* (PP). CK = control.

No.	AS	GZ	PO	PC	SS	BC	RS	FO	CA	PP
**2a**	37.5	12.5	10.0	29.0	38.5	7.7	30.8	20.0	13.3	20.0
**2b**	43.8	33.3	50.0	32.3	84.6	53.8	50.0	20.0	46.7	48.0
**2c**	25.0	25.0	20.0	12.9	38.5	46.2	11.5	15.0	20.0	36.0
**2d**	25.0	29.2	40.0	32.3	69.2	53.8	38.5	10.0	33.3	44.0
**2e**	56.3	29.2	20.0	38.7	80.8	26.9	11.5	45.0	33.3	72.0
**2f**	37.5	25.0	40.0	58.1	88.5	50.0	38.5	20.0	46.7	76.0
**2g**	43.8	8.3	10.0	25.8	38.5	15.4	80.8	35.0	26.7	36.0
**2h**	18.8	16.7	20.0	22.6	46.2	23.1	38.5	15.0	13.3	40.0
**2i**	25.0	4.2	20.0	25.8	53.8	30.8	50.0	20.0	26.7	24.0
**2j**	25.0	16.7	8.0	32.3	46.2	11.5	46.2	45.0	26.7	36.0
**2k**	25.0	12.5	20.0	48.4	84.6	38.5	38.5	20.0	33.3	76.0
**2l**	12.5	4.2	8.0	32.3	50.0	26.9	11.5	20.0	20.0	28.0
**2m**	18.8	4.2	8.0	16.1	57.7	19.2	19.2	15.0	13.3	36.0
**2n**	37.5	33.3	20.0	29.0	84.6	57.7	69.2	35.0	60.0	76.0
**2o**	37.5	12.5	8.0	25.8	53.8	23.1	38.5	15.0	20.0	8.0
**2p**	43.8	25.0	40.0	45.2	57.7	42.3	76.9	25.0	26.7	28.0
FP	83.3	85.4	63.6	58.1	75.0	56.7	69.7	42.9	37.5	65.4
CK	0.0	0.0	0.0	0.0	0.0	0.0	0.0	0.0	0.0	0.0

## Data Availability

Data are contained within the article or Supplementary Material.

## Supplementary Materials

The following supporting information can be downloaded at: https://www.mdpi.com/article/10.3390/molecules28083373/s1, Figures for ^1^H NMR, ^13^C NMR, and HRMS.

## Sample Availability

Samples of the compounds are available from the authors.
