# Supplementary material for: Synthesis, Structural Determination, and Antifungal Activity of Novel Fluorinated Quinoline Analogs
Source: Molecules. 2023 Apr 11;28(8):3373. doi: 10.3390/molecules28083373 (PMC10145707; doi:10.3390/molecules28083373)
Supplement: Supplementary file 1 [file molecules-28-03373-s001.zip › molecules-2332406-supplementary.pdf]

Supplemental Materials

Synthesis, Crystal Structure and Antifungal Activity of Novel Fluorinated Quinoline Derivatives

Xin-Peng Sun[1,3], Wei Yu[1,3], Li-Jing Min[2], Liang Han[3], Xue-Wen Hua[4], Jian-Jun Shi[5], Na-Bo Sun[1*], Xing-Hai Liu[3*]

[1] College of Biology and Environmental Engineering, Zhejiang Shuren University, Hangzhou 310015, Zhejiang, China

[2] College of Life Science, Key Laboratory of Vector Biology and Pathogen Control of Zhejiang Province, Huzhou University, Huzhou, 313000, Zhejiang, China

[3] College of Chemical Engineering, Zhejiang University of Technology, Hangzhou, 310014, Zhejiang, China;

[4] College of Agriculture, Liaocheng University, Liaocheng, Shandong 252000, China

[5] College of Chemistry and Chemical Engineering, Huangshan University, Huangshan, 245041, China

Figures of contents

1. The spectrocopy of target compounds………..................... 2-10

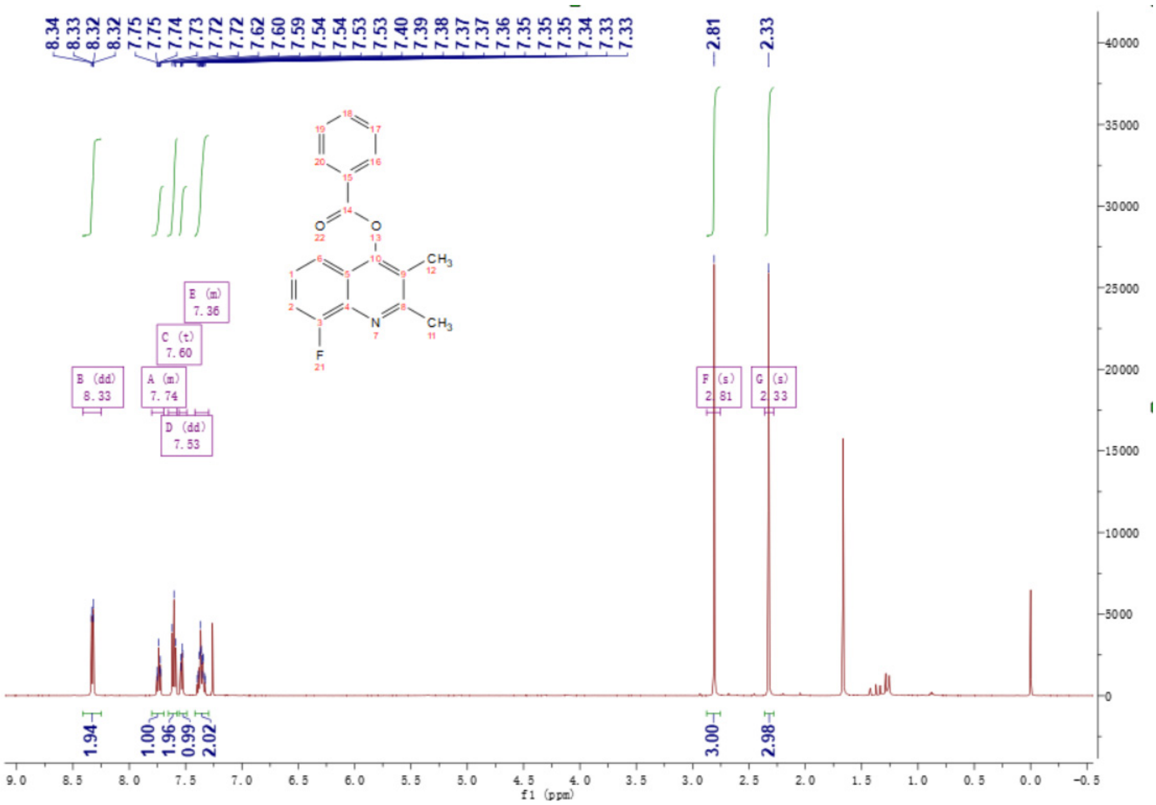

^{1}H NMR of compound 2a

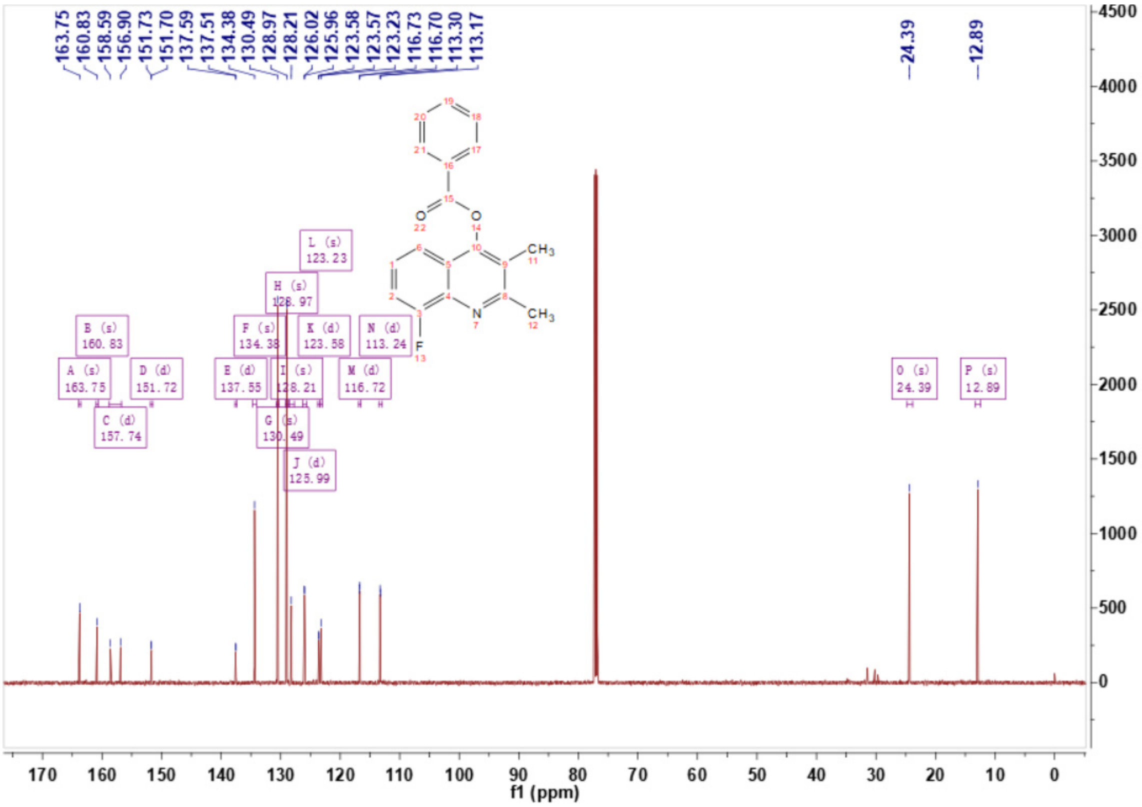

^{13}C NMR of compound 2a

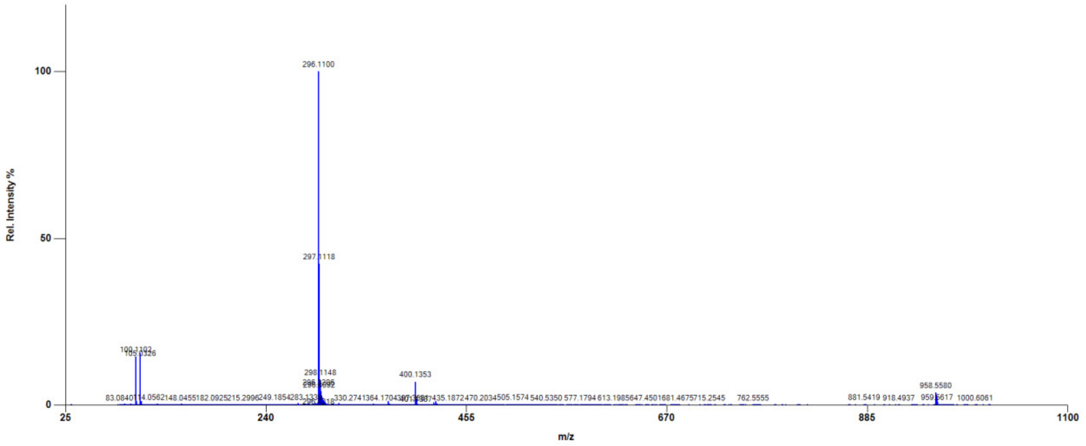

HRMS of compound 2a

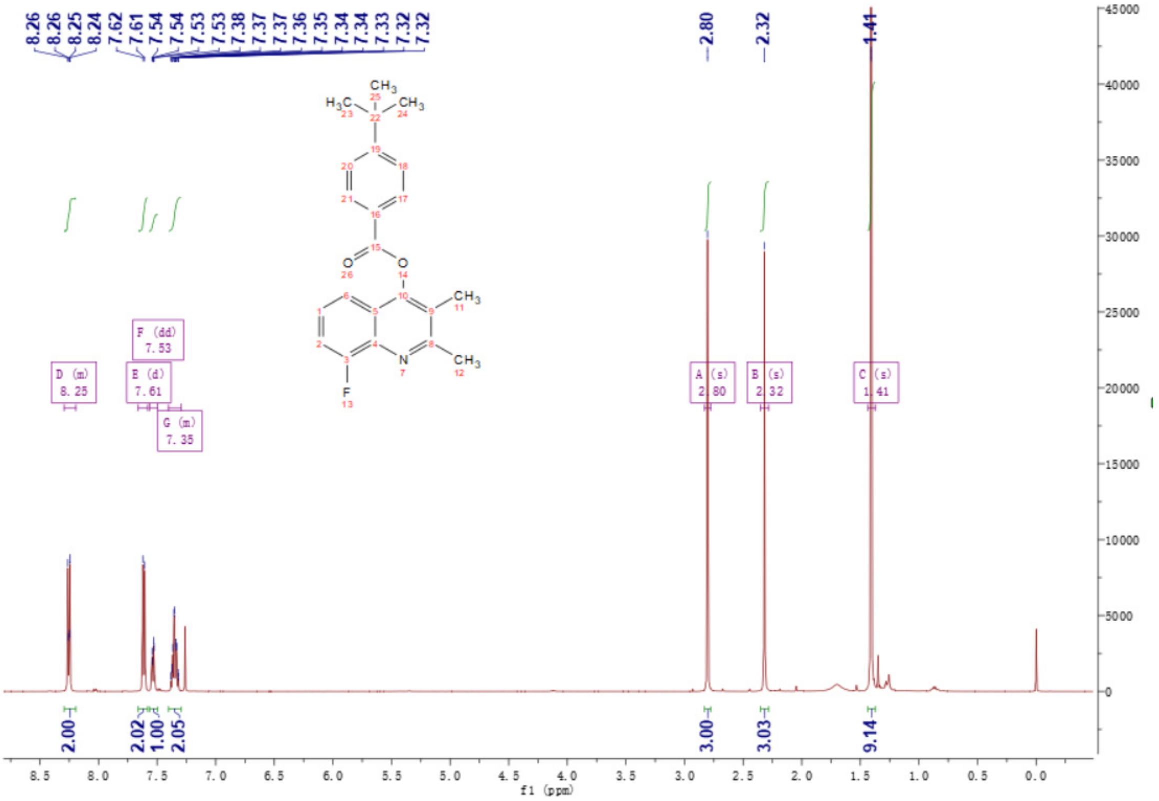

^{1}H NMR of compound 2b

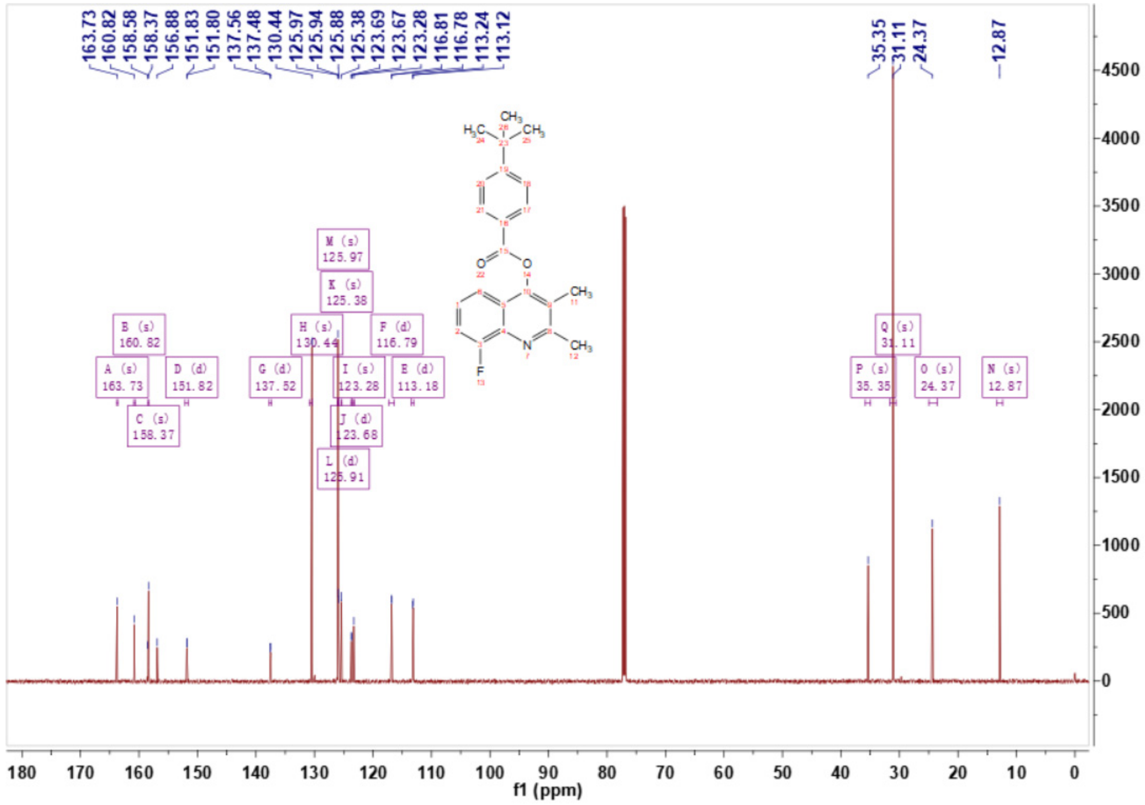

^{13}C NMR of compound 2b

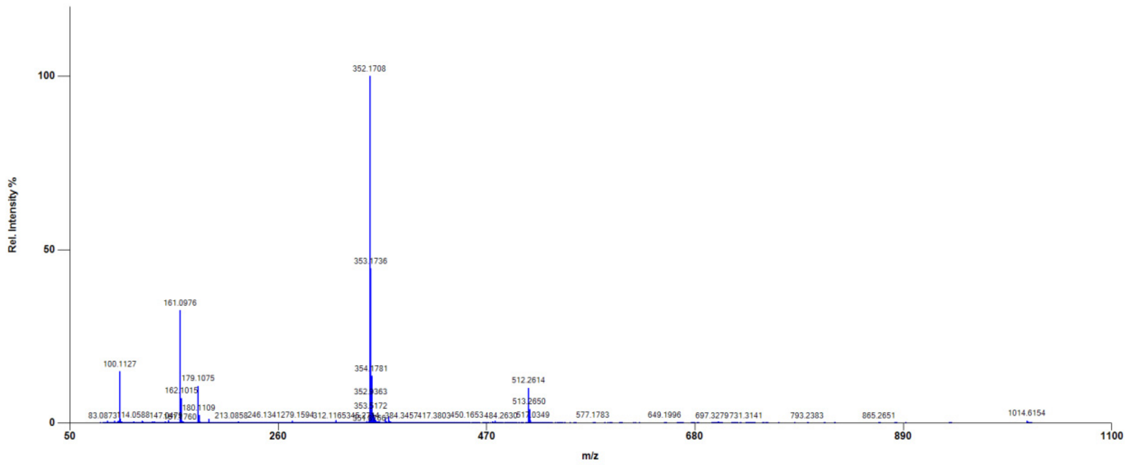

HRMS of compound 2b

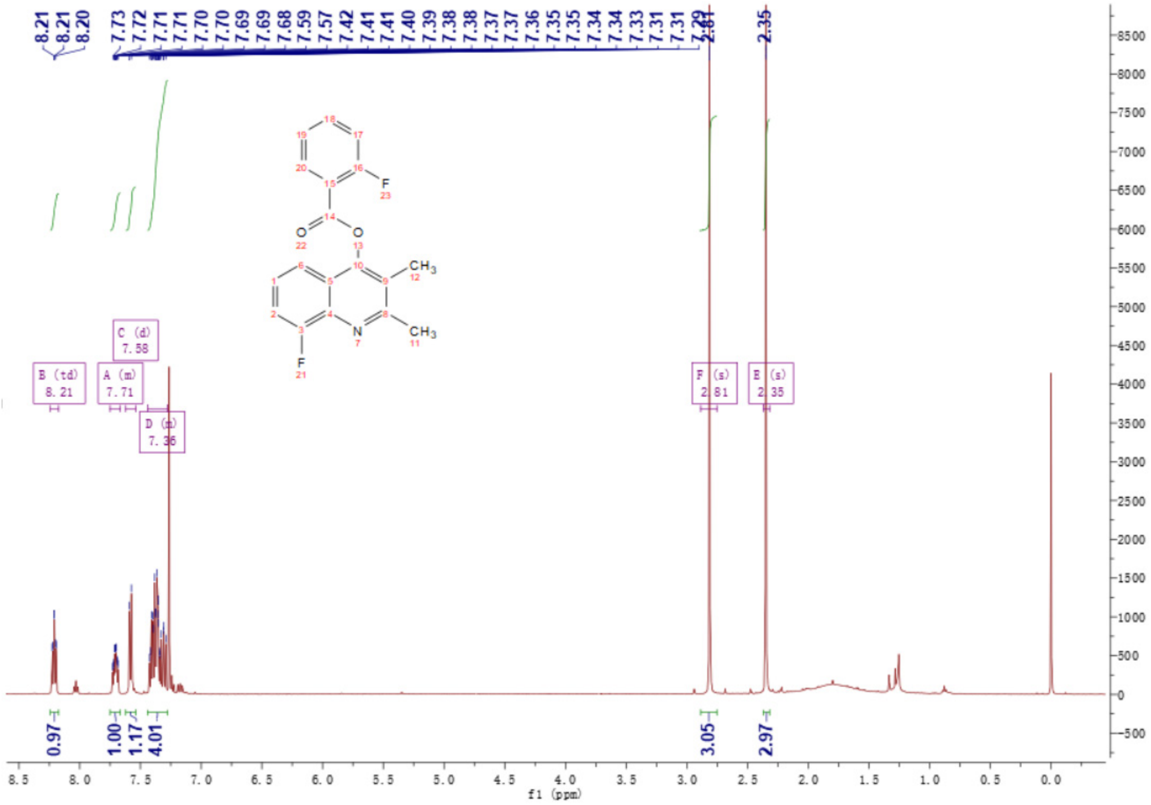

^1H NMR of compound 2c

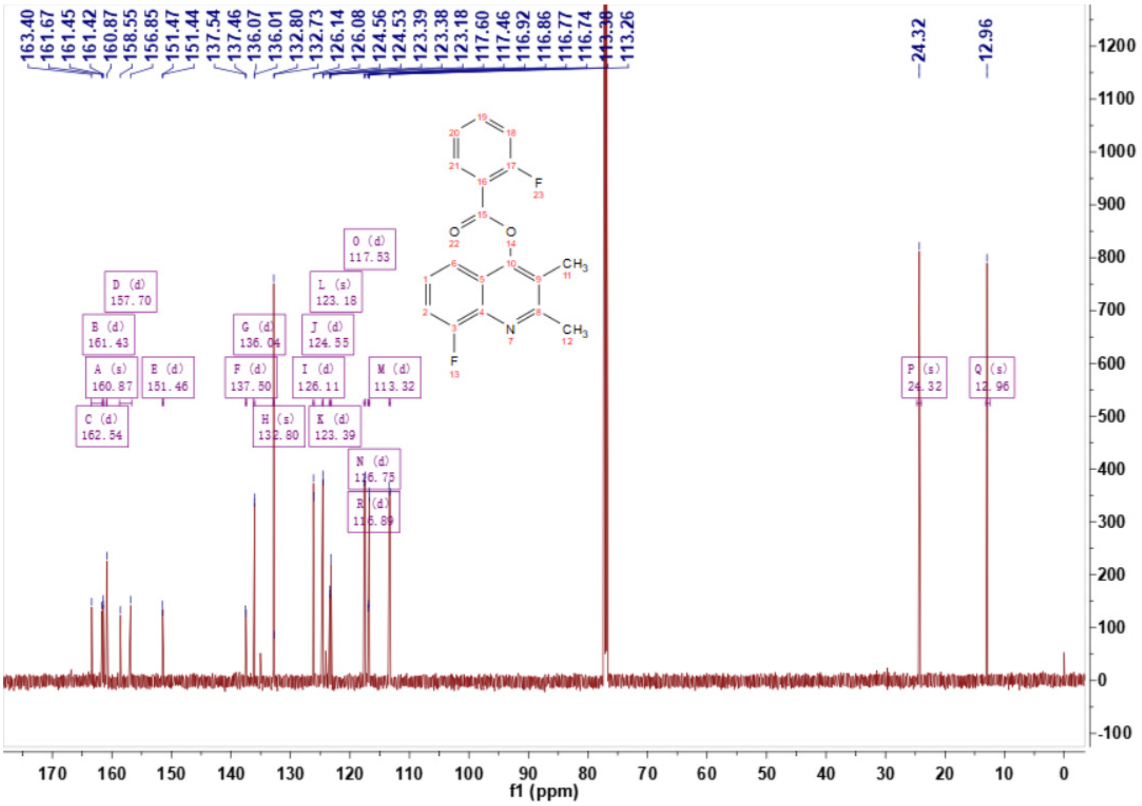

^{13}C NMR of compound 2c

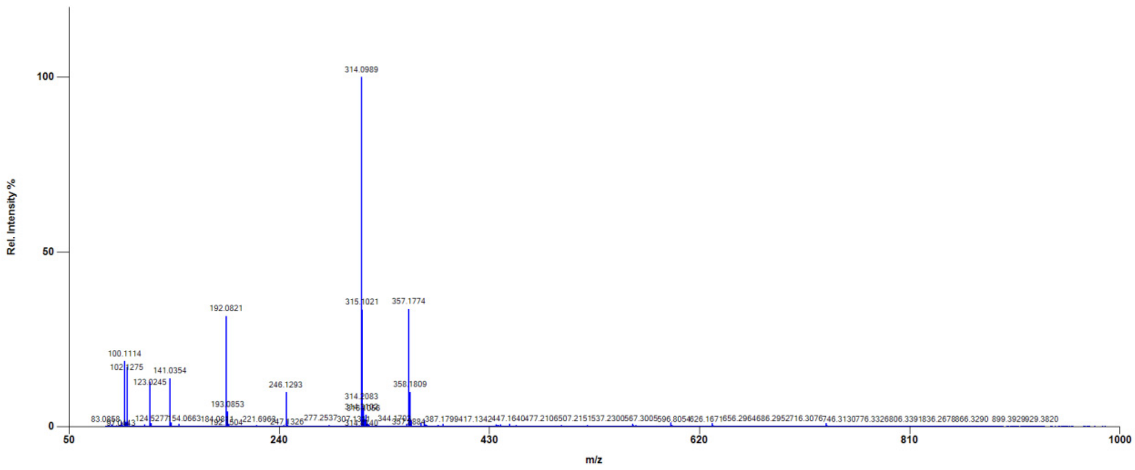

HRMS of compound 2c

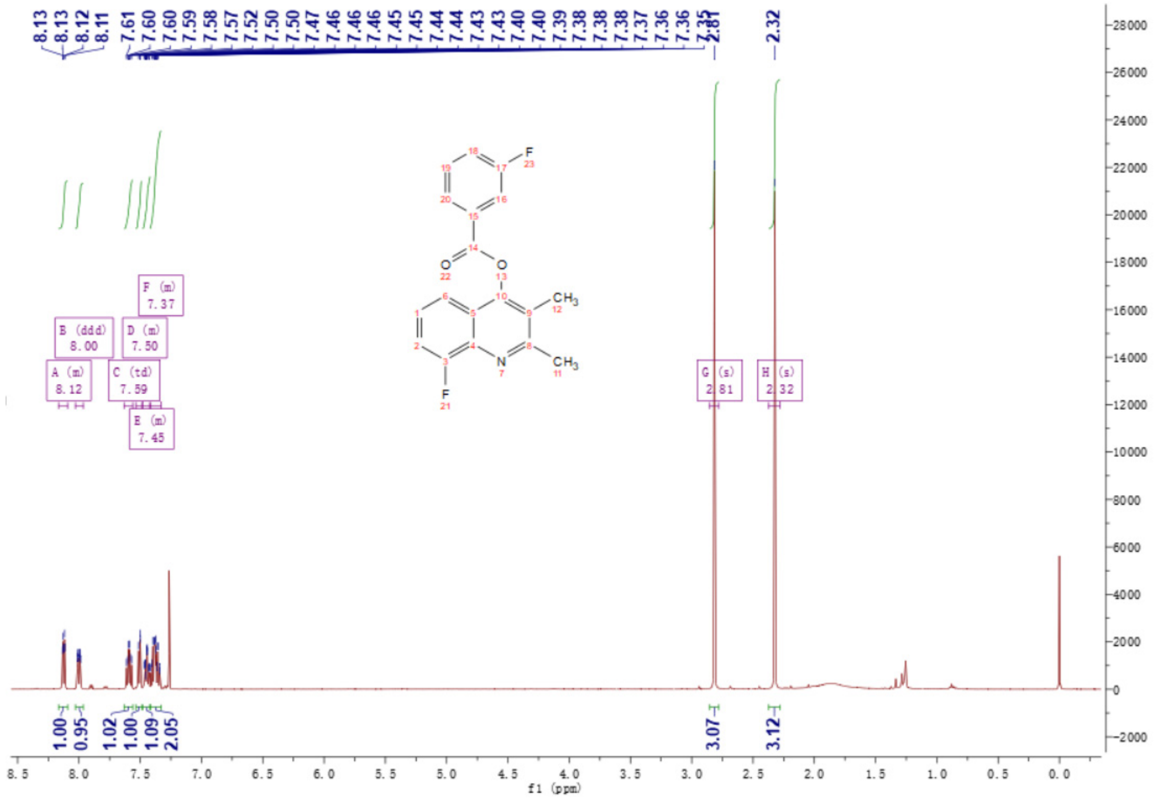

^{1}H NMR of compound 2d

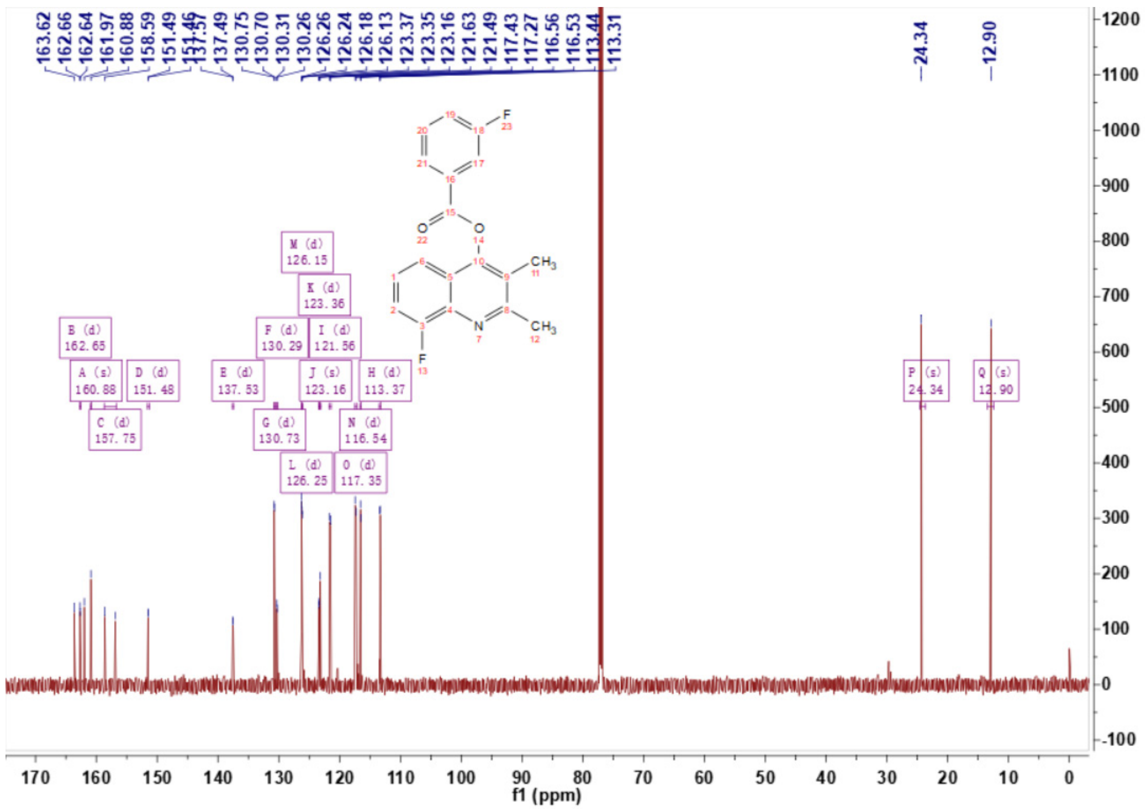

^{13}C NMR of compound 2d

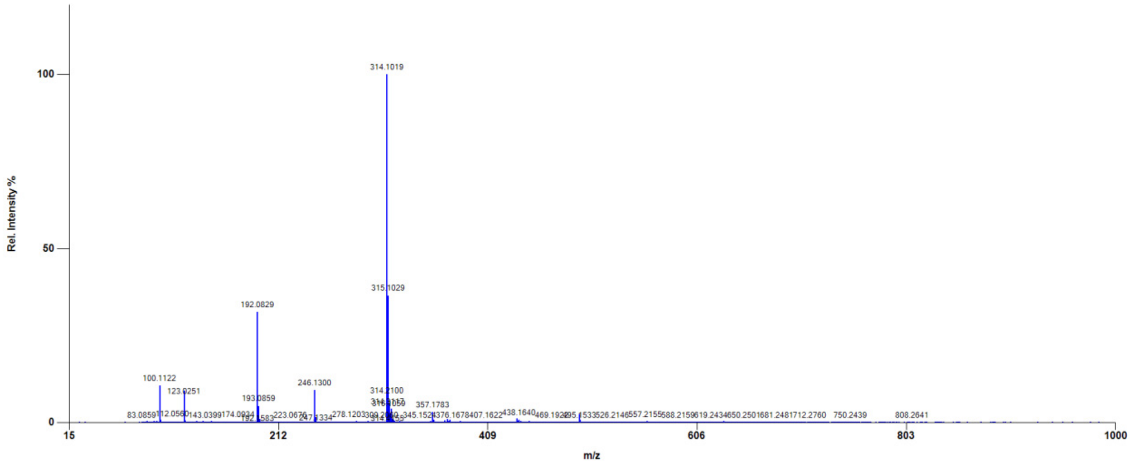

HRMS of compound 2d

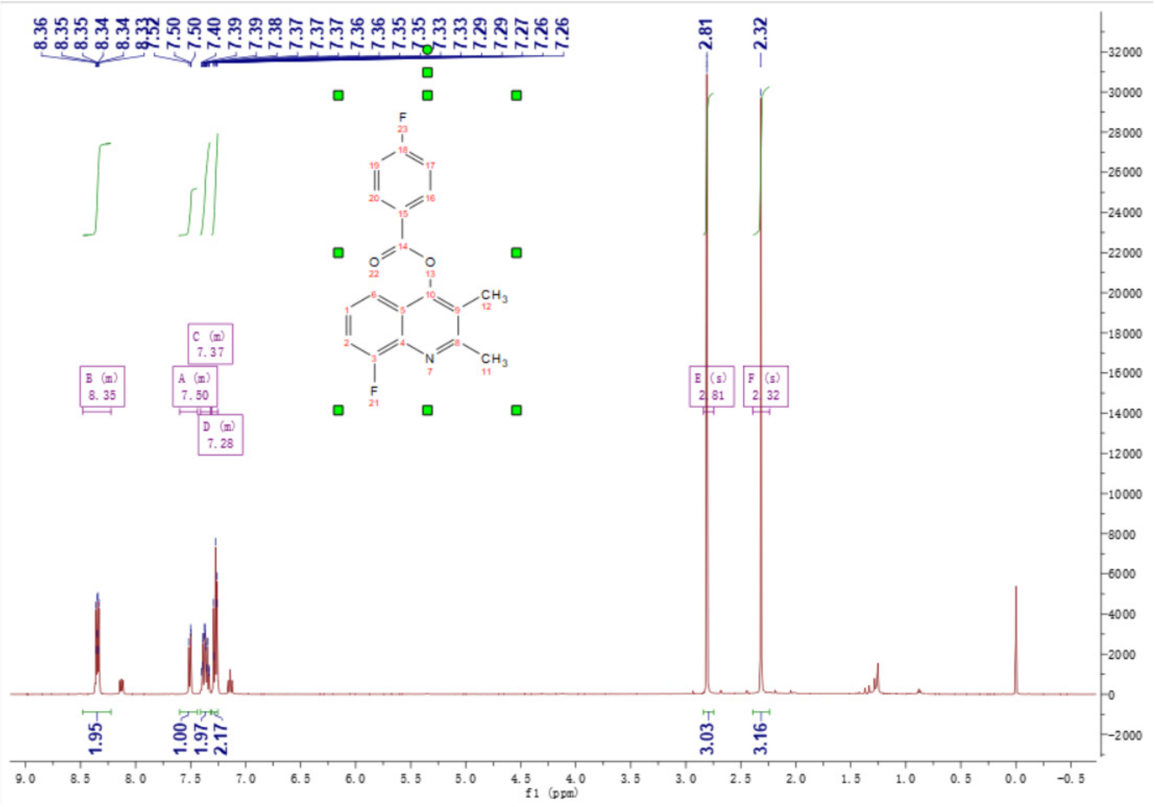

^{1}H NMR of compound 2e

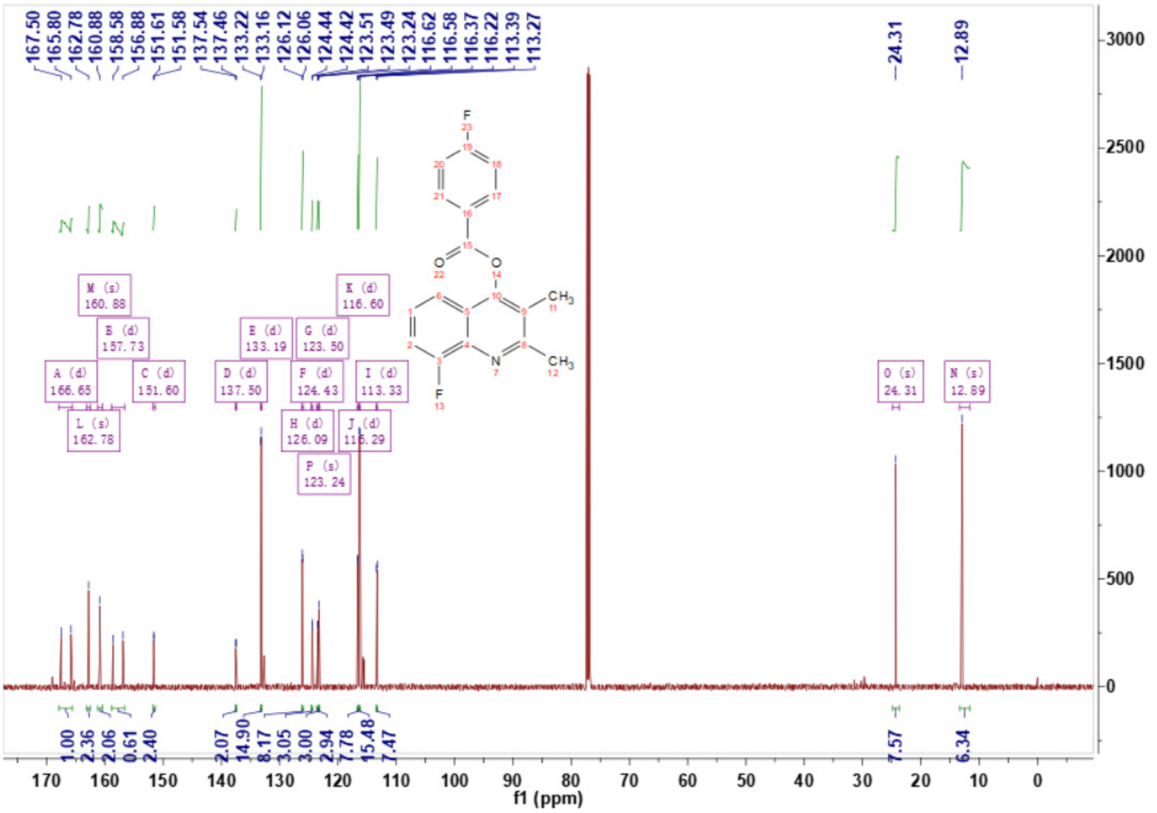

^{13}C NMR of compound 2e

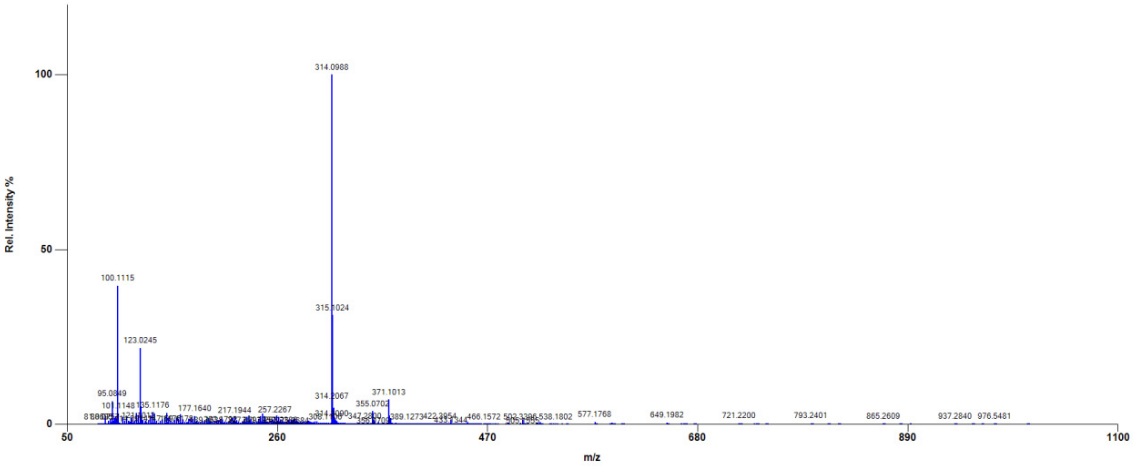

HRMS of compound 2e

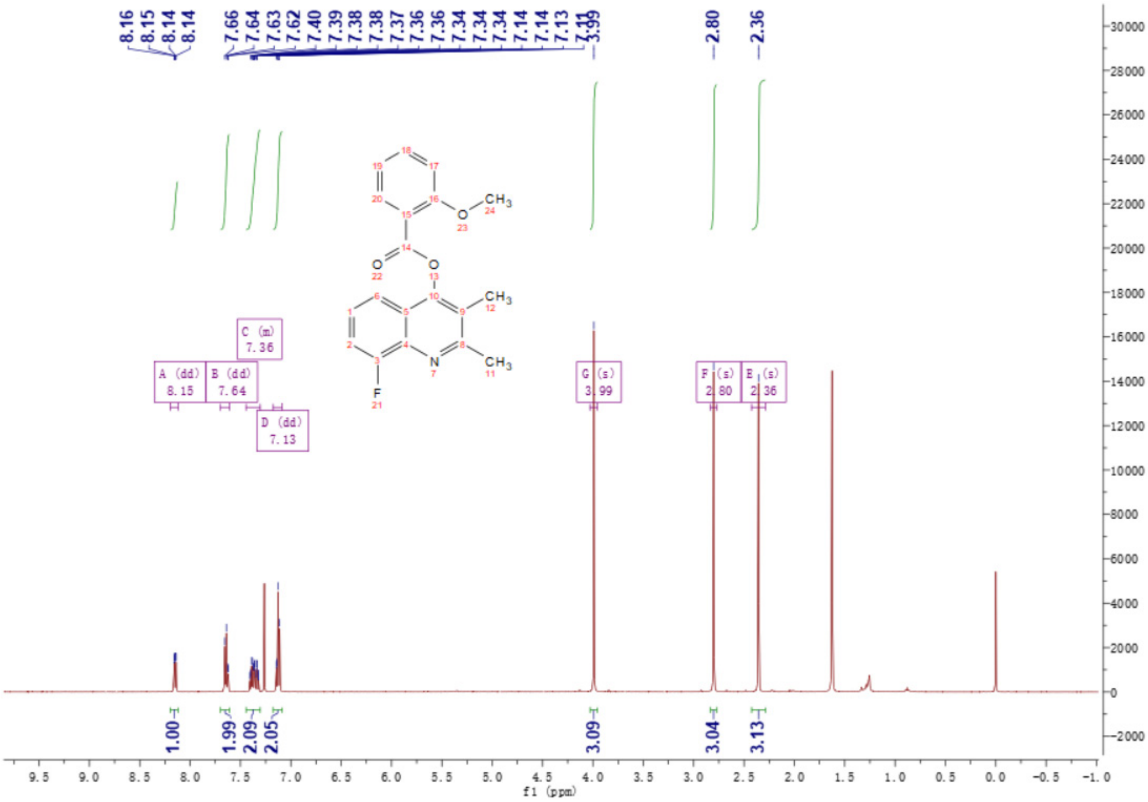

^{1}H NMR of compound 2f

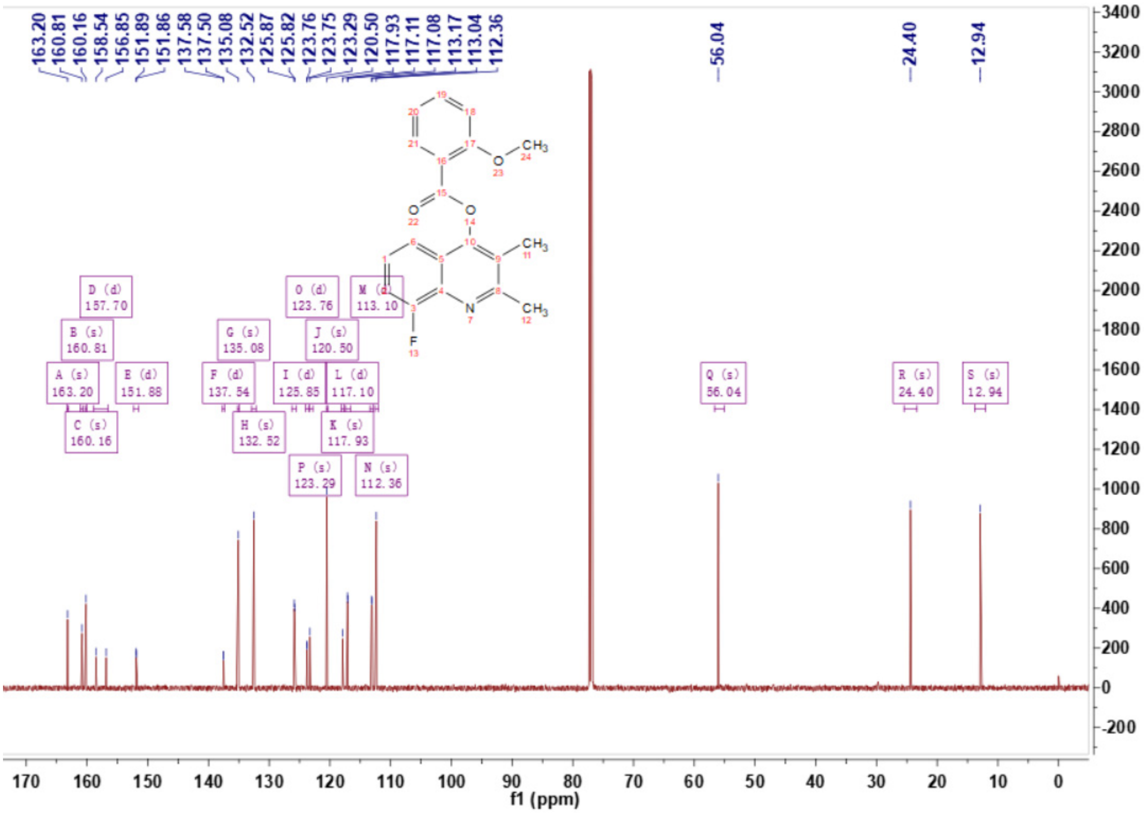

^{13}C NMR of compound 2f

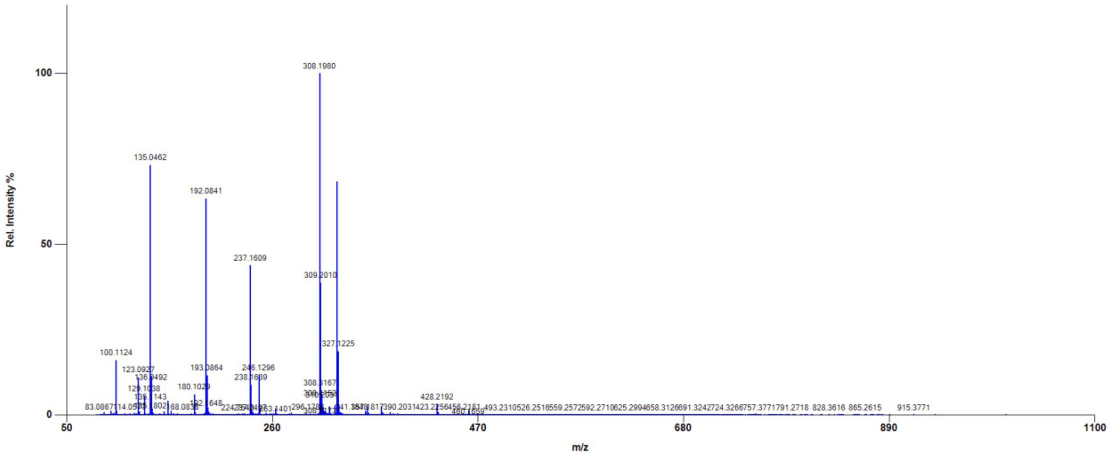

HRMS of compound 2f

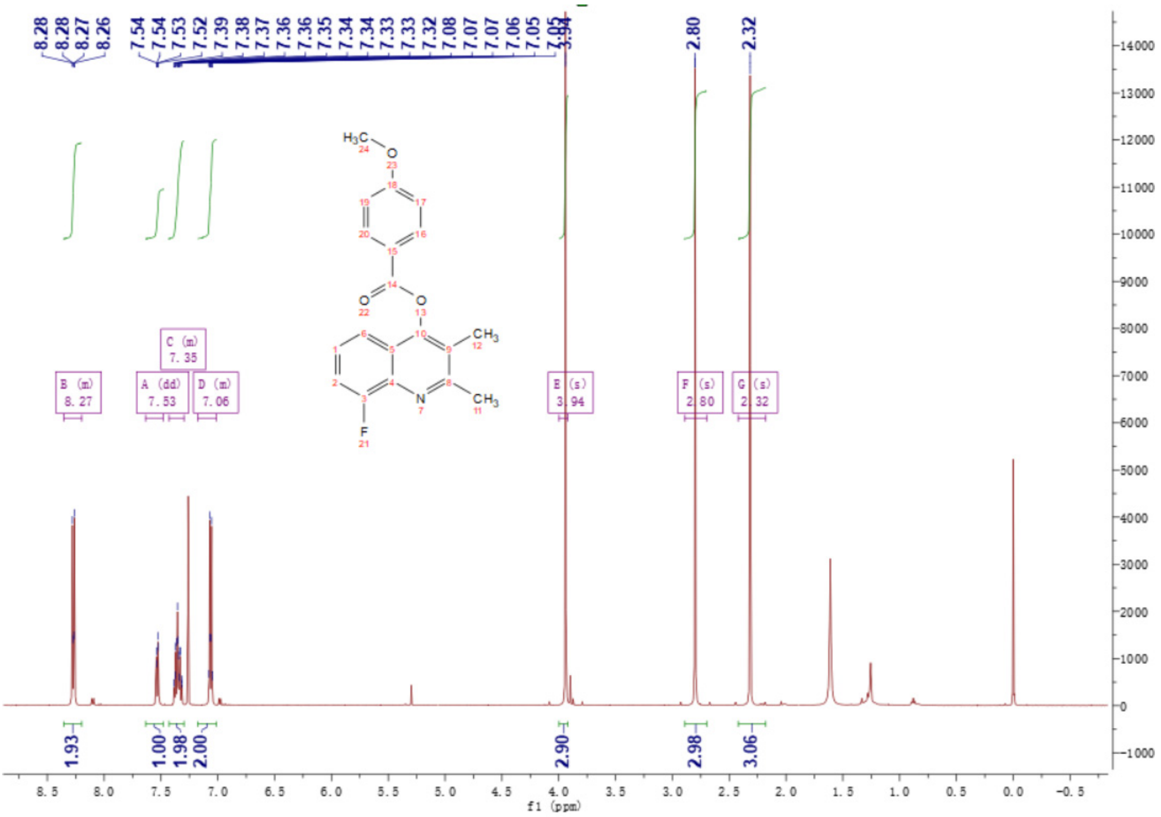

^{1}H NMR of compound 2g

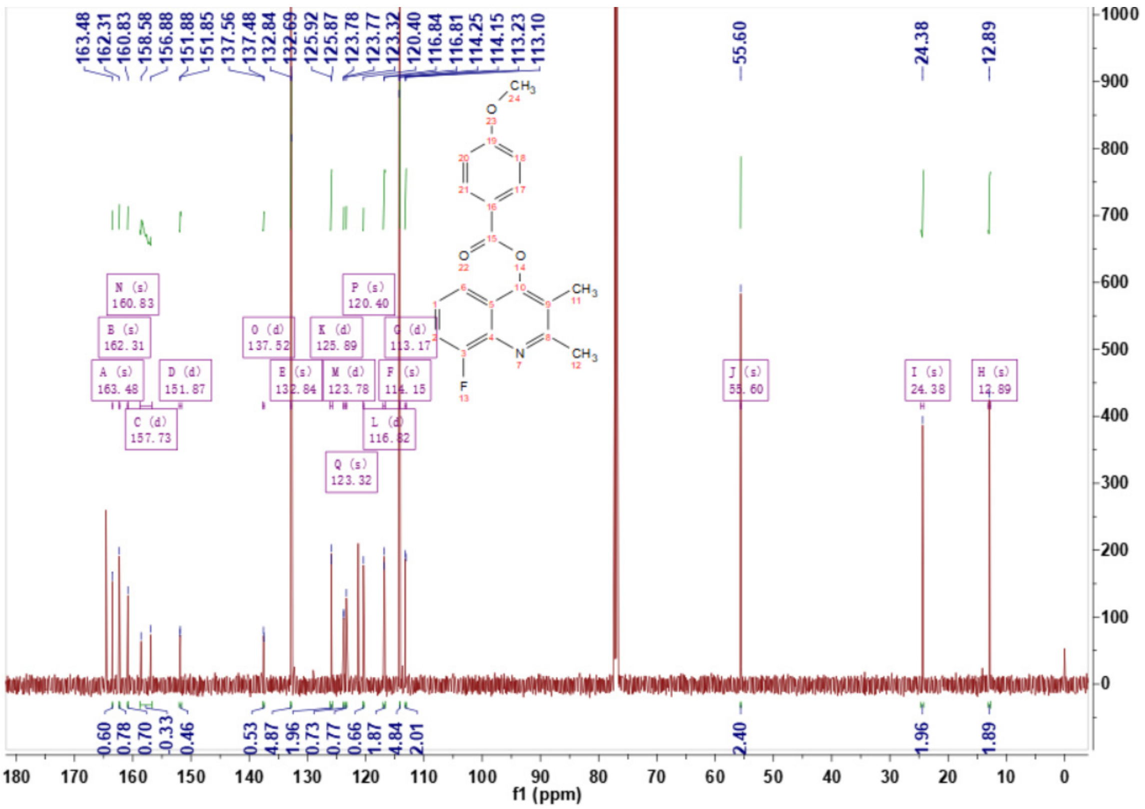

^{13}C NMR of compound 2g

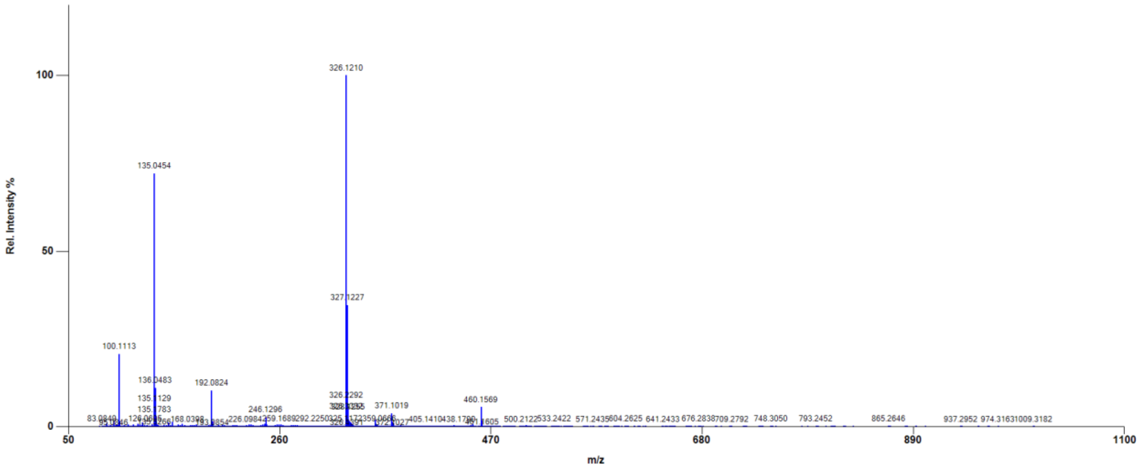

HRMS of compound 2g

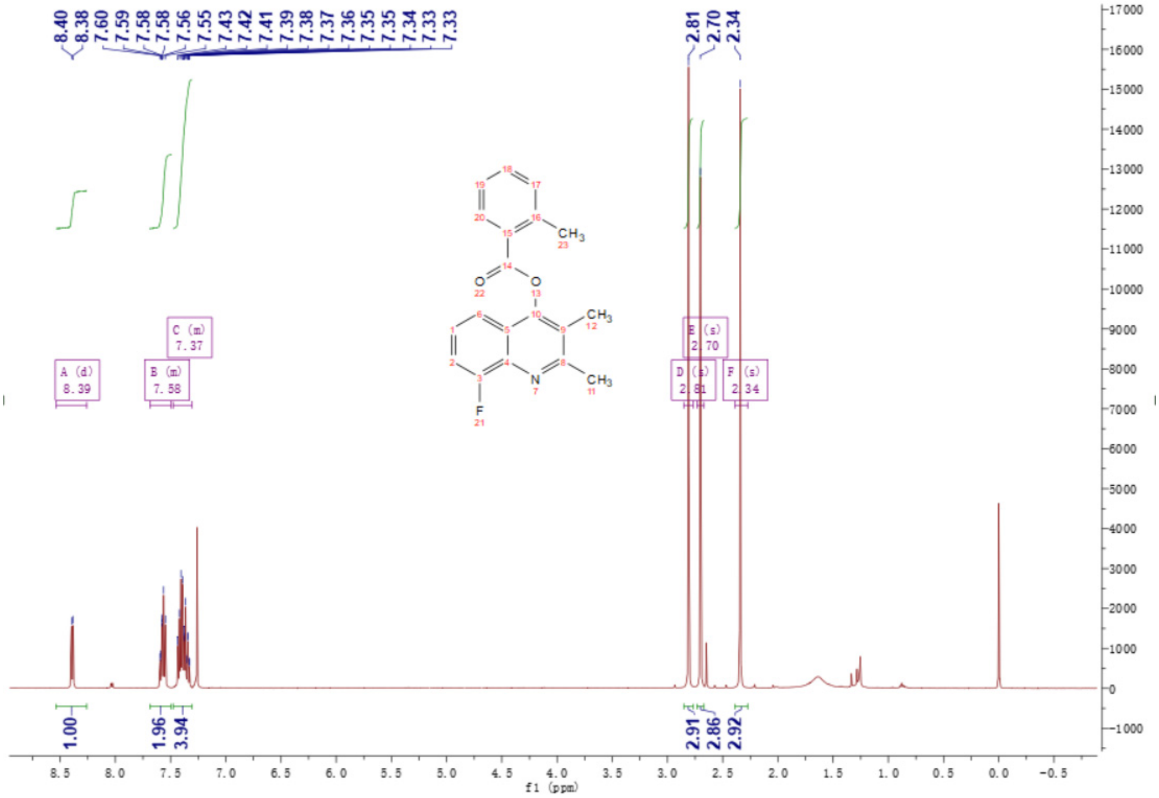

^{1}H NMR of compound 2h

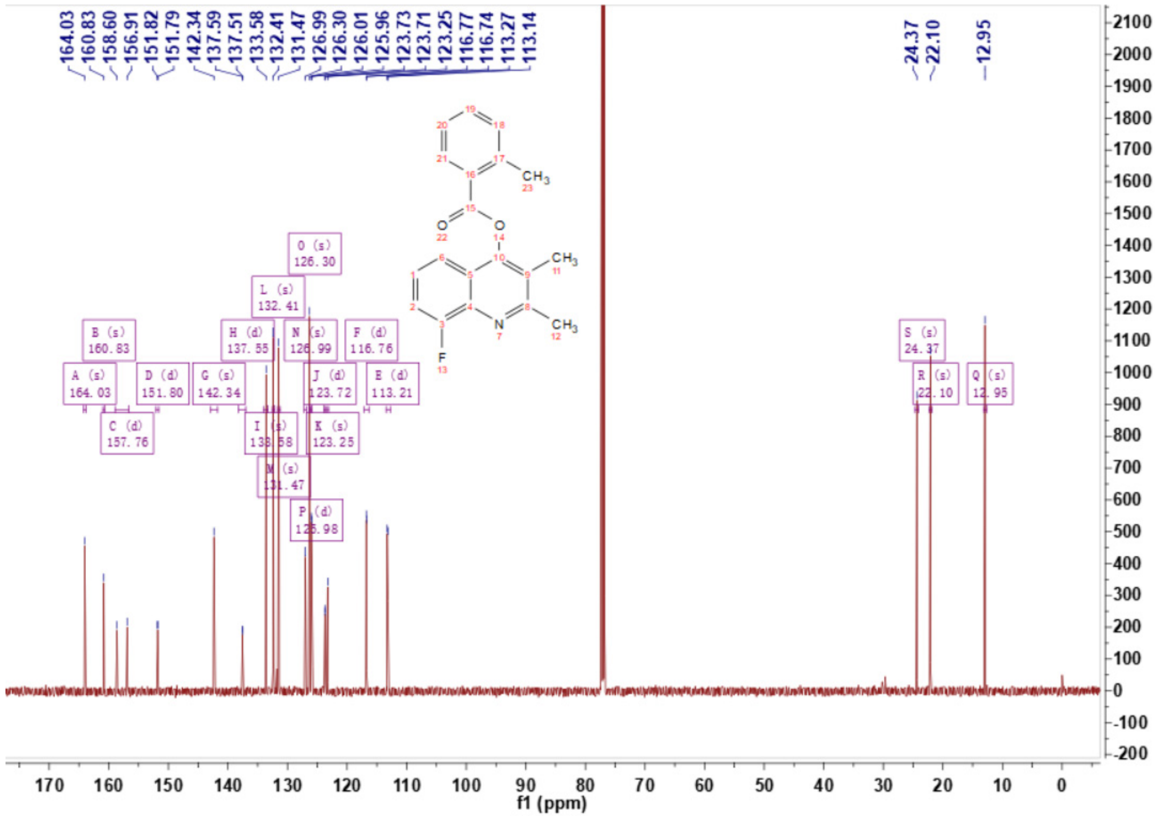

^{13}C NMR of compound 2h

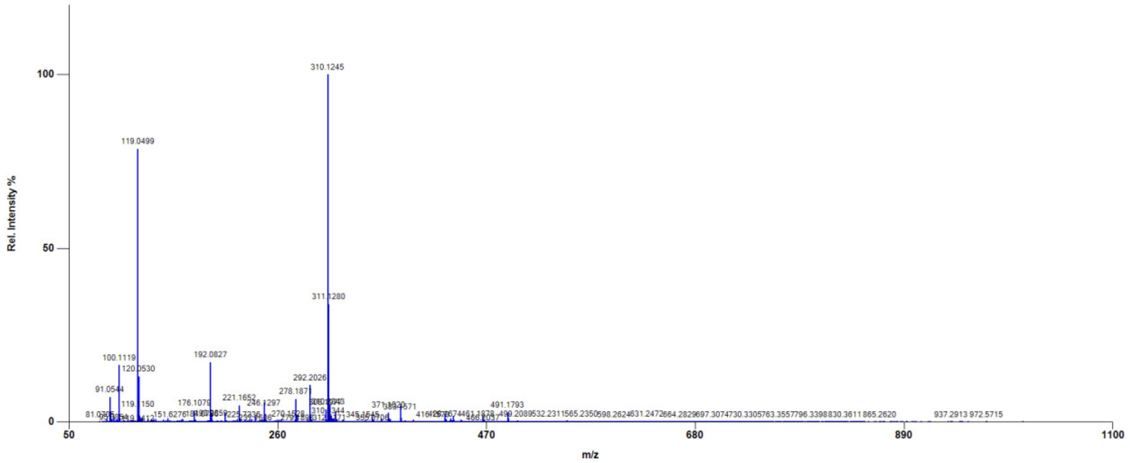

HRMS of compound 2h

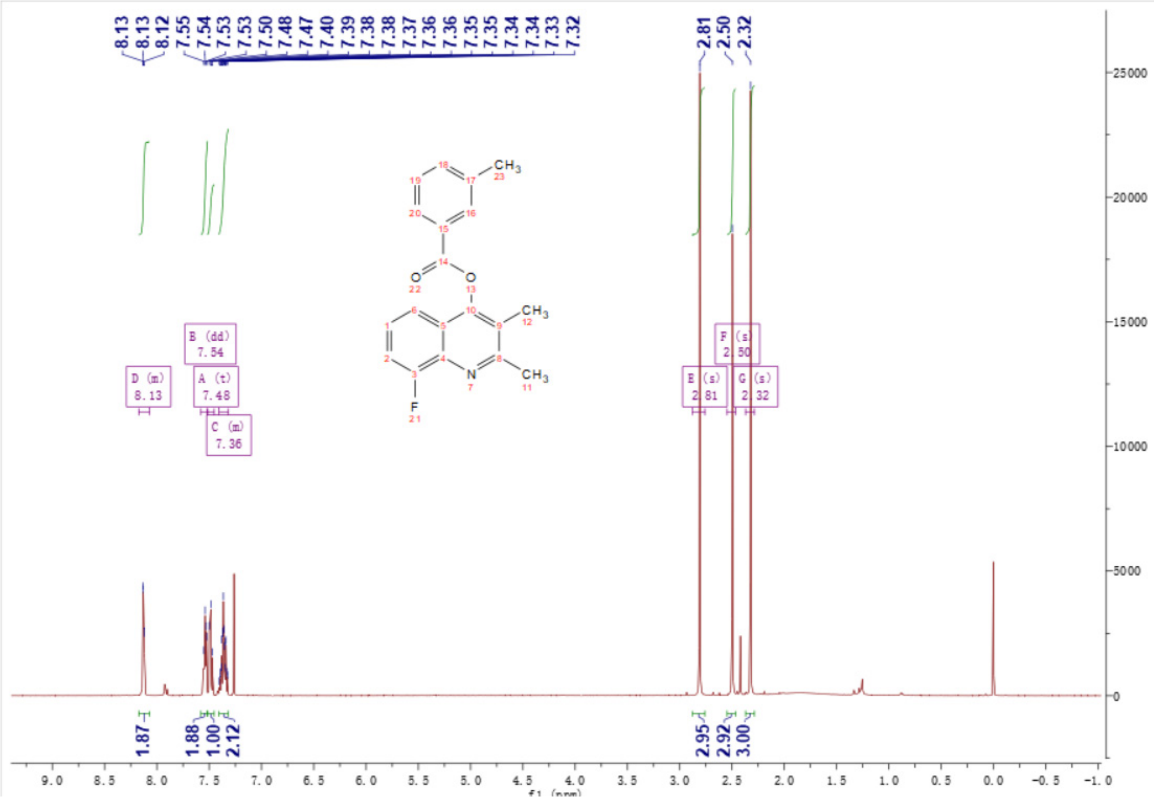

^{1}H NMR of compound 2i

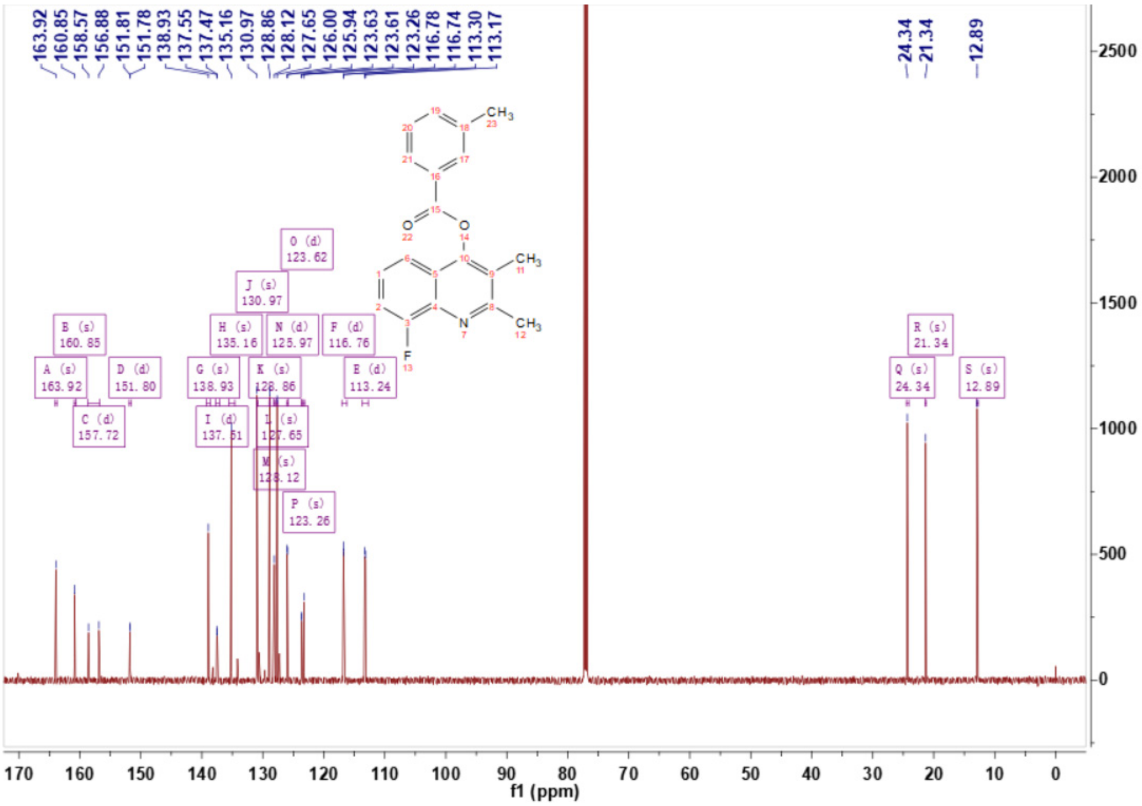

^{13}C NMR of compound 2i

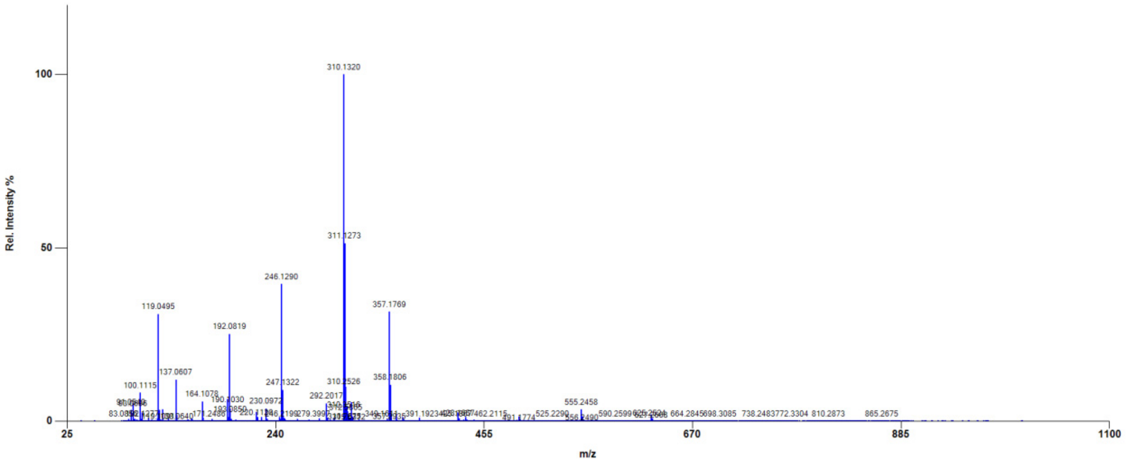

HRMS of compound 2i

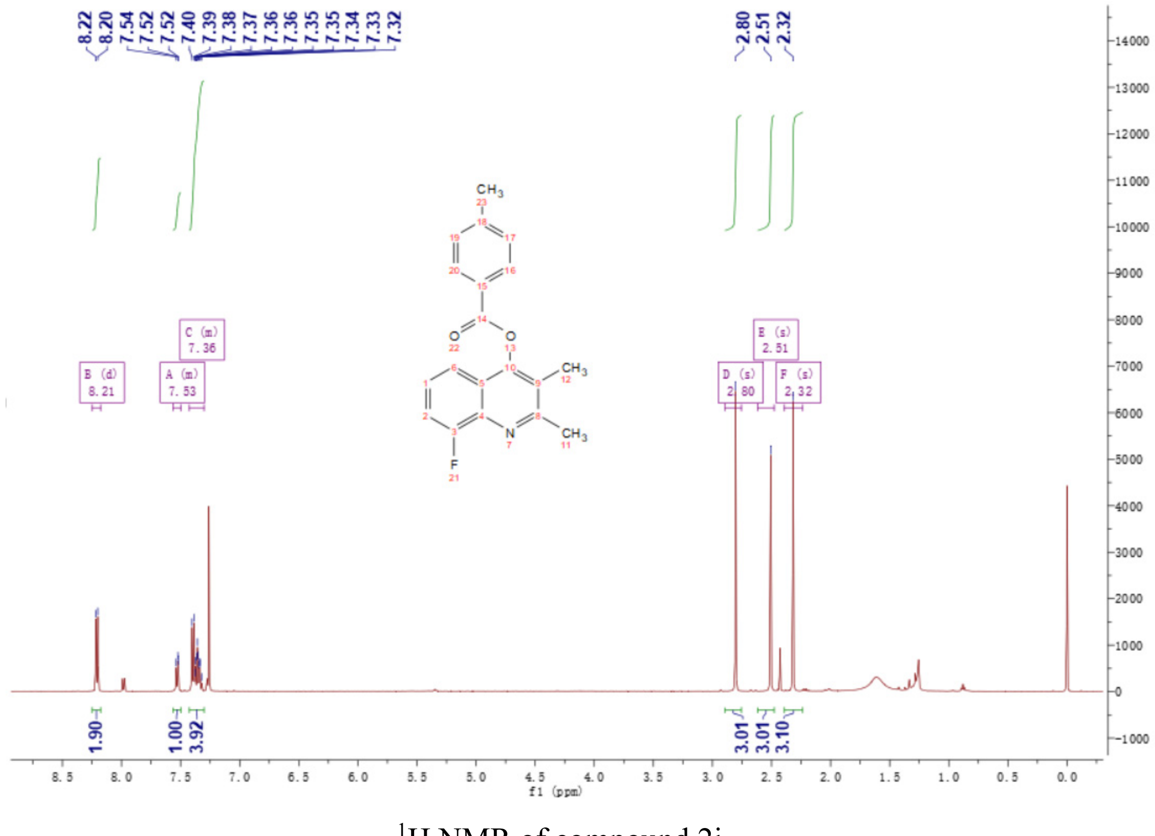

^{1}H NMR of compound 2j

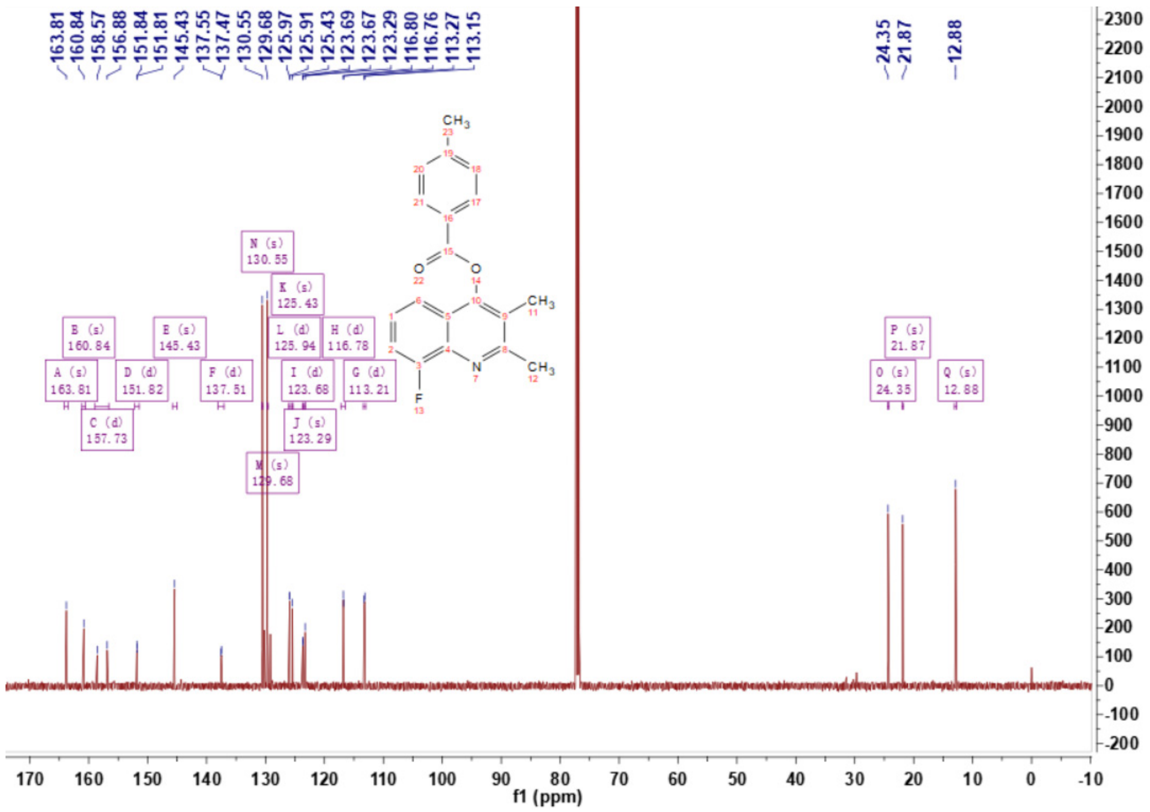

^{13}C NMR of compound 2j

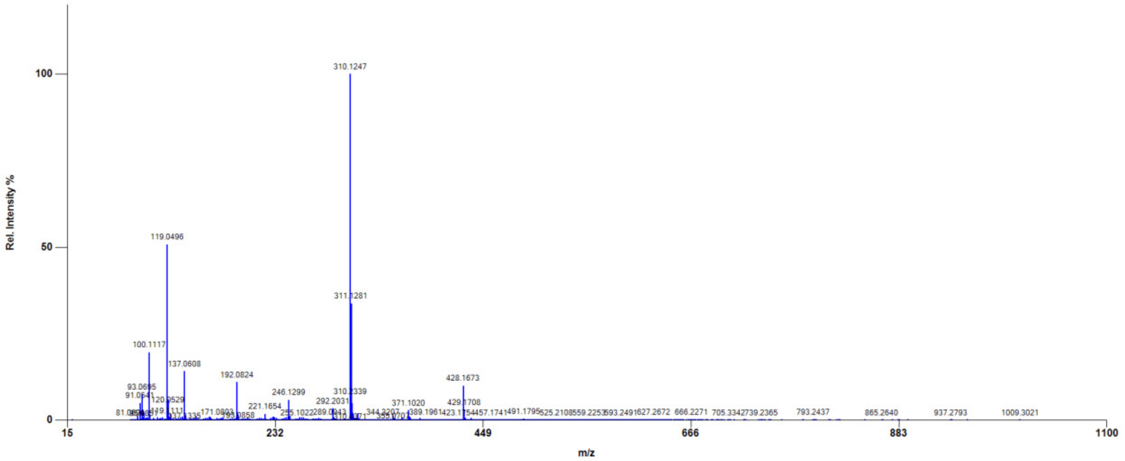

HRMS of compound 2j

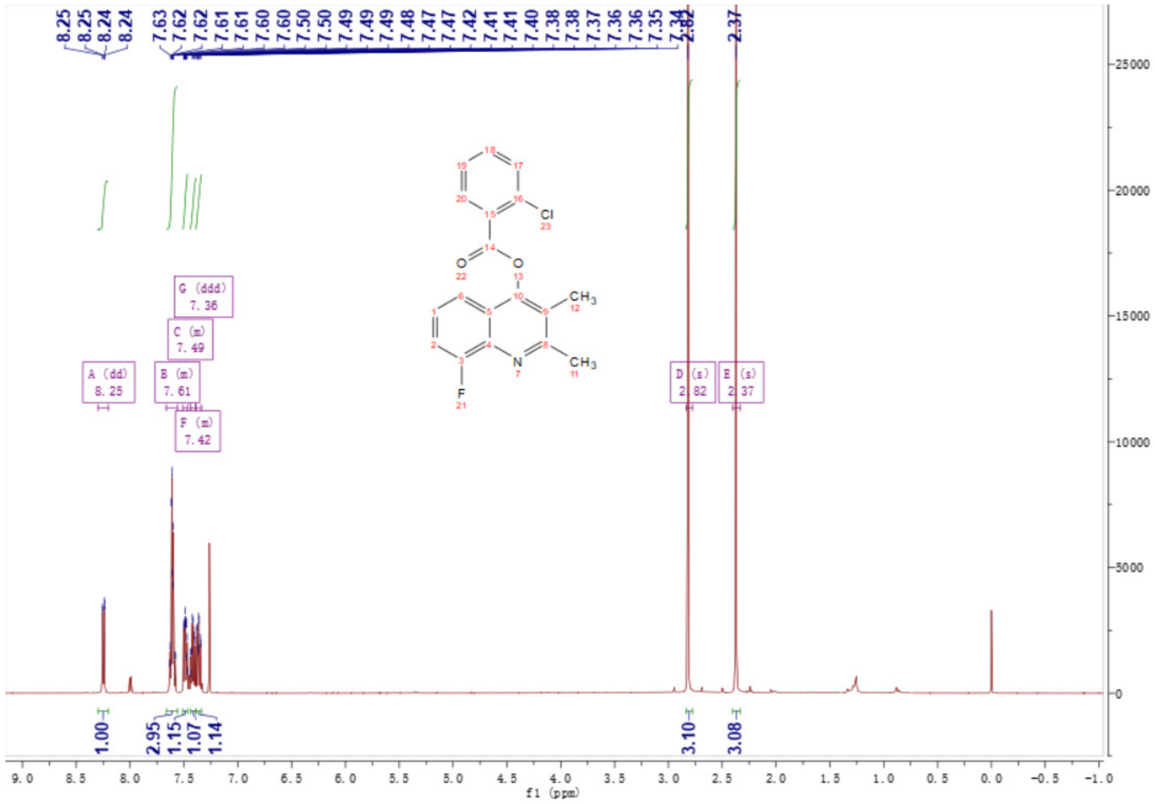

^{1}H NMR of compound 2k

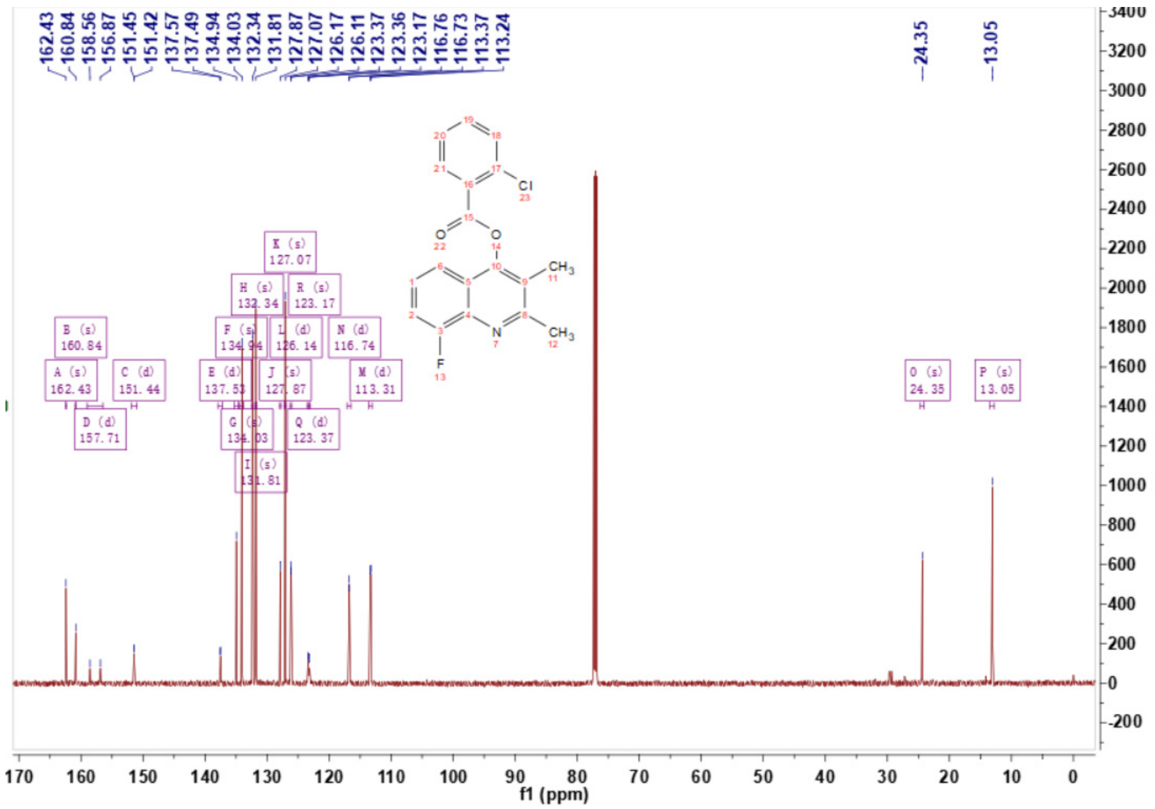

^{13}C NMR of compound 2k

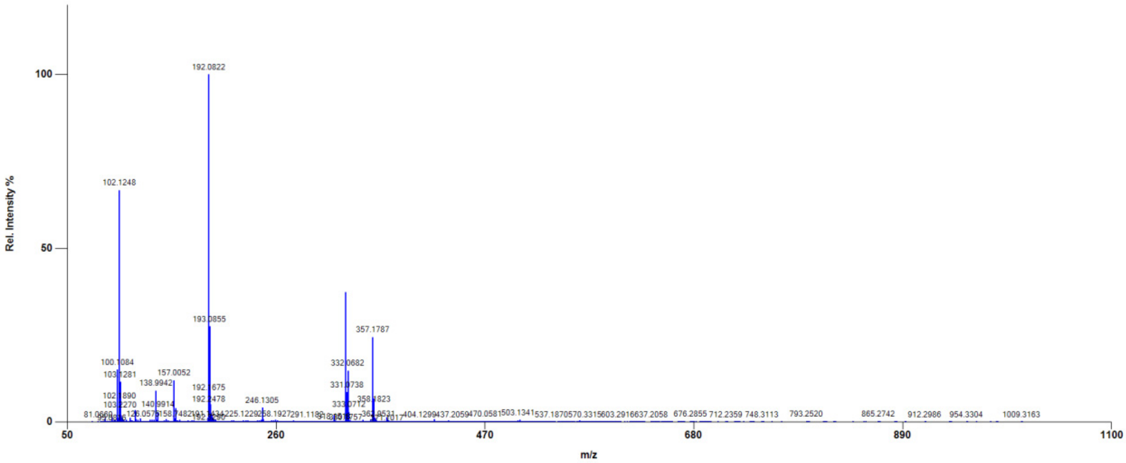

HRMS of compound 2k

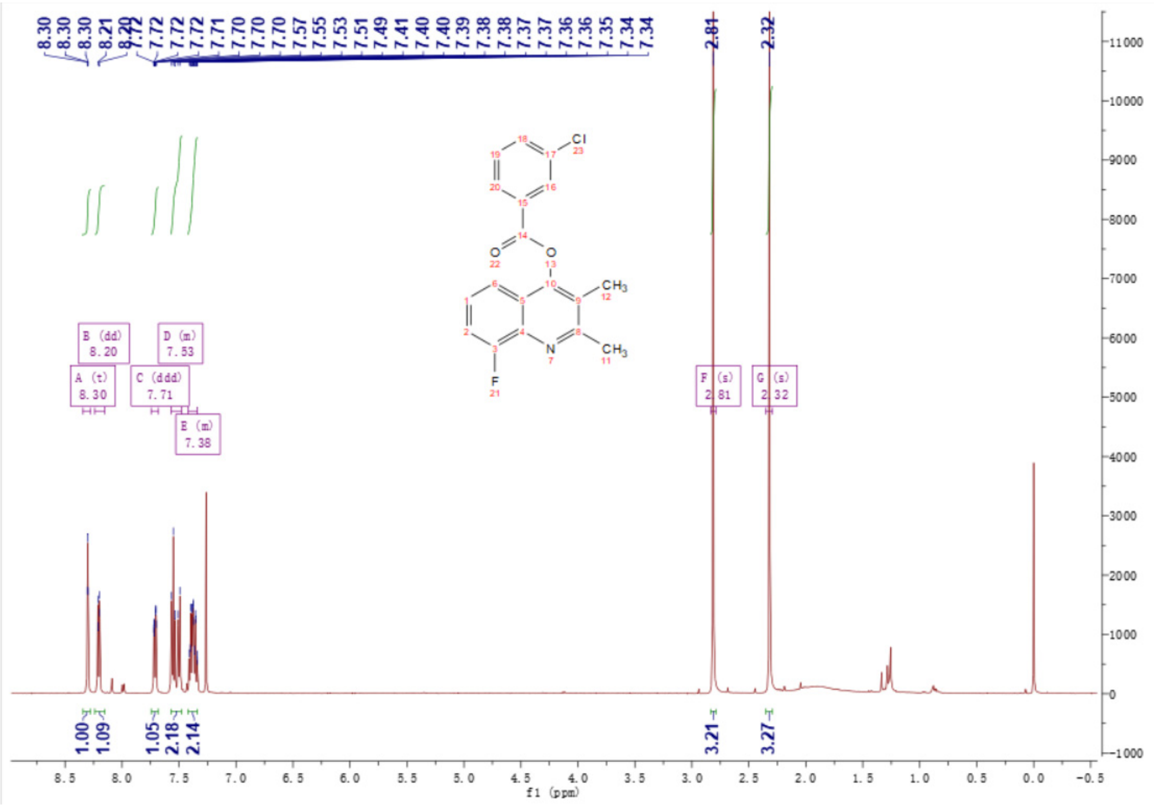

^{1}H NMR of compound 2l

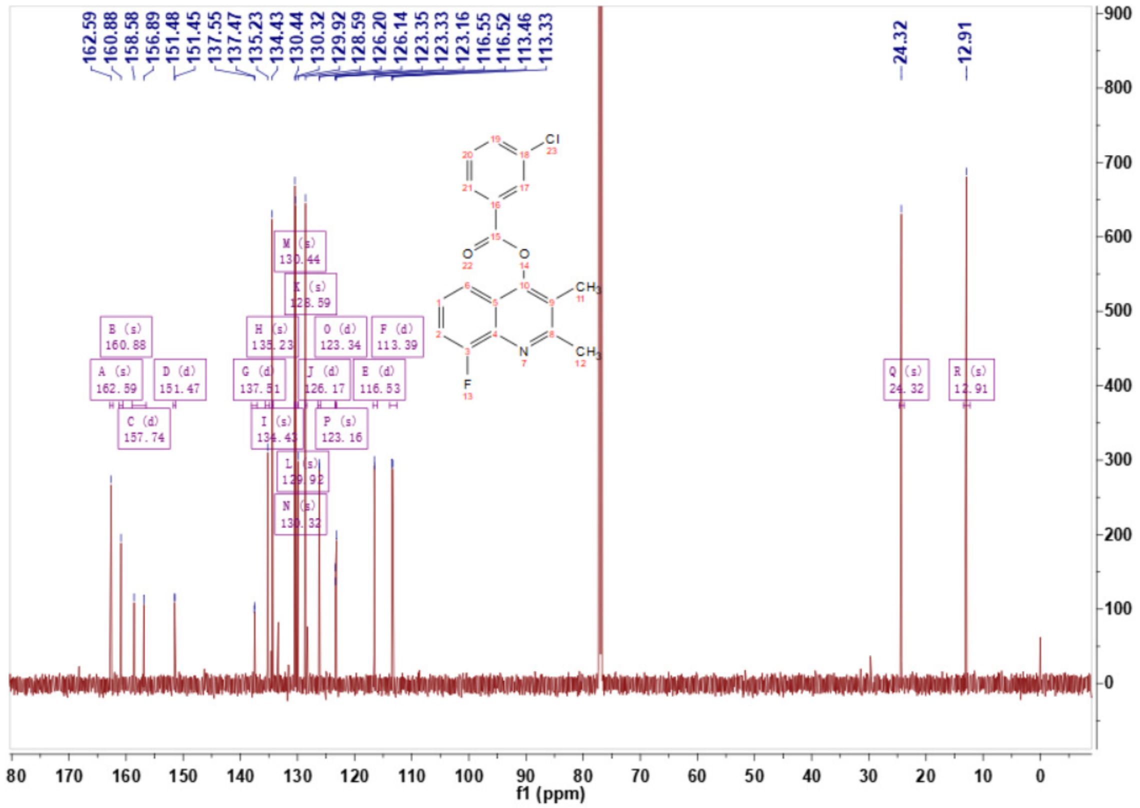

^{13}C NMR of compound 2l

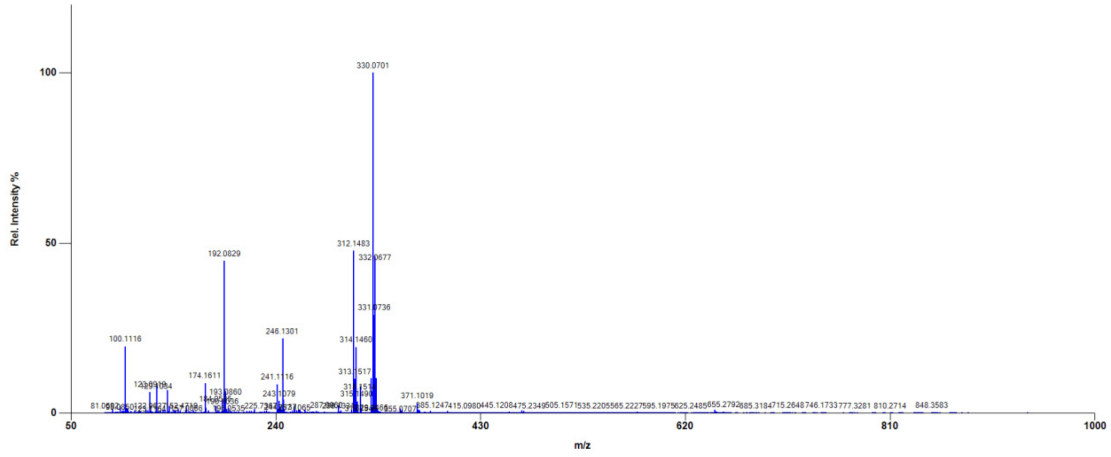

HRMS of compound 2l

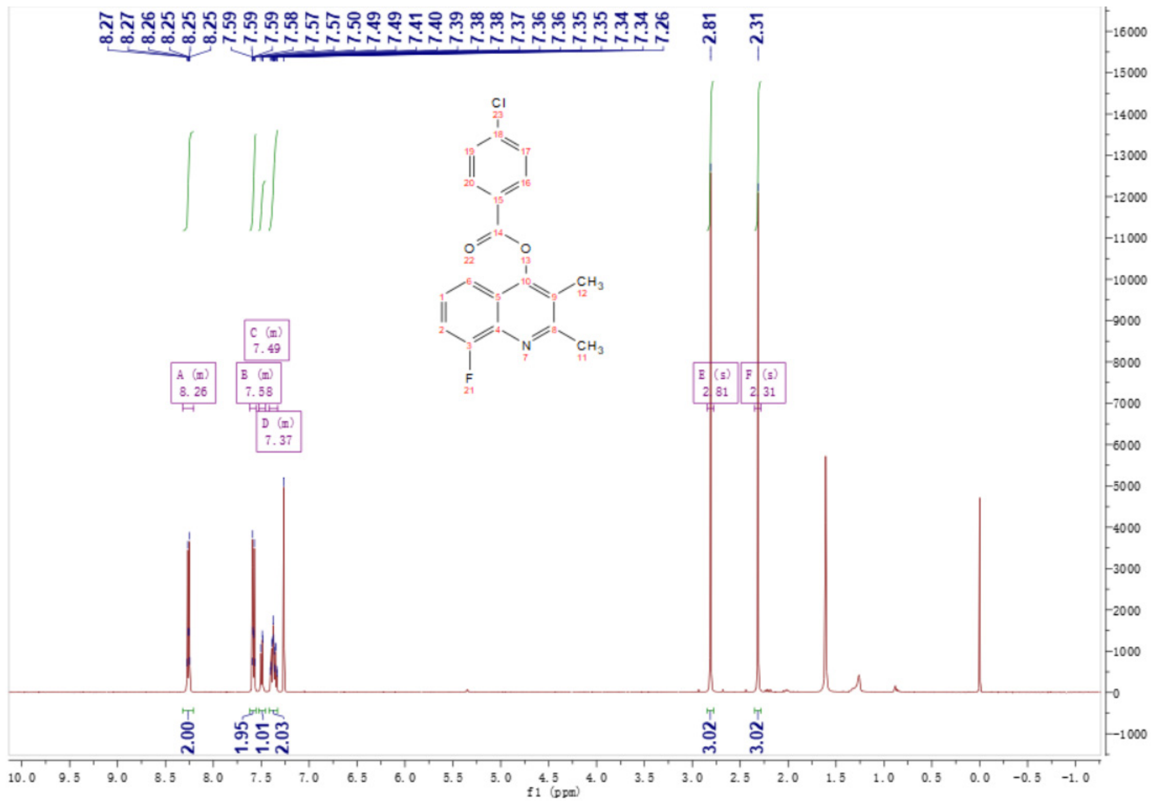

^{1}H NMR of compound 2m

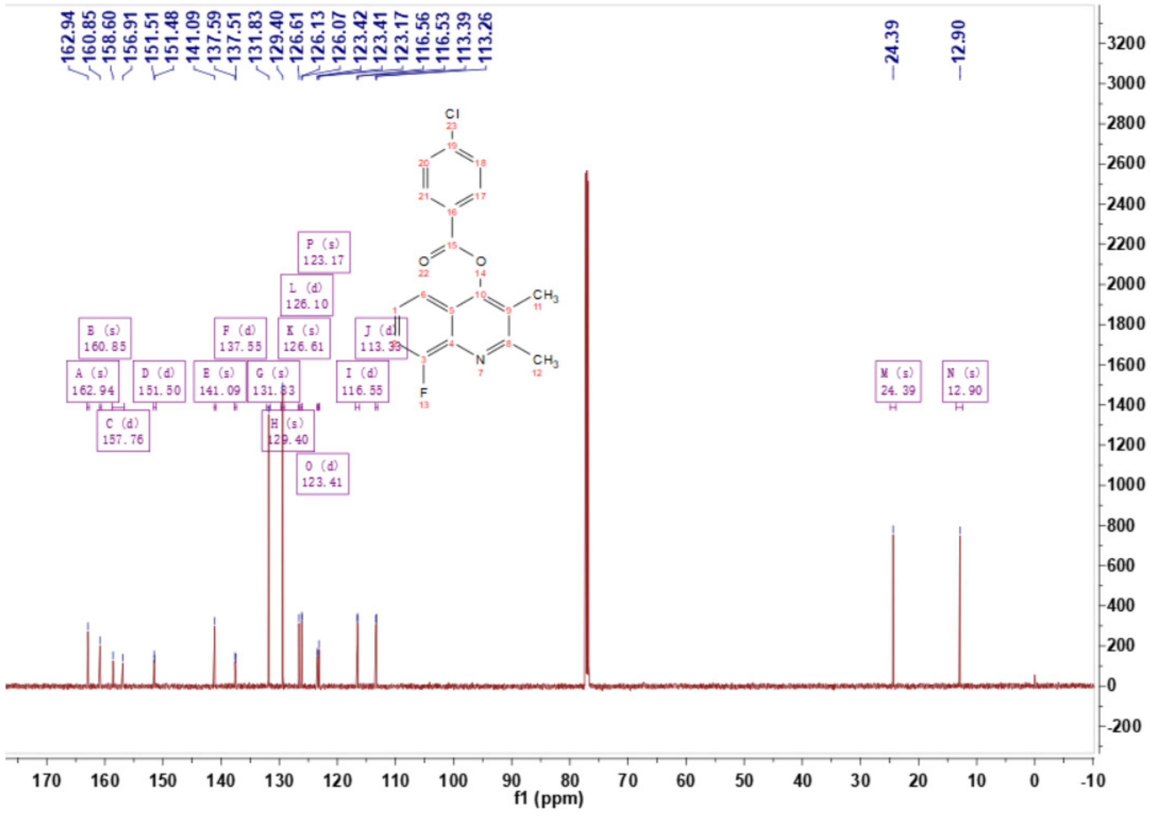

^{13}C NMR of compound 2m

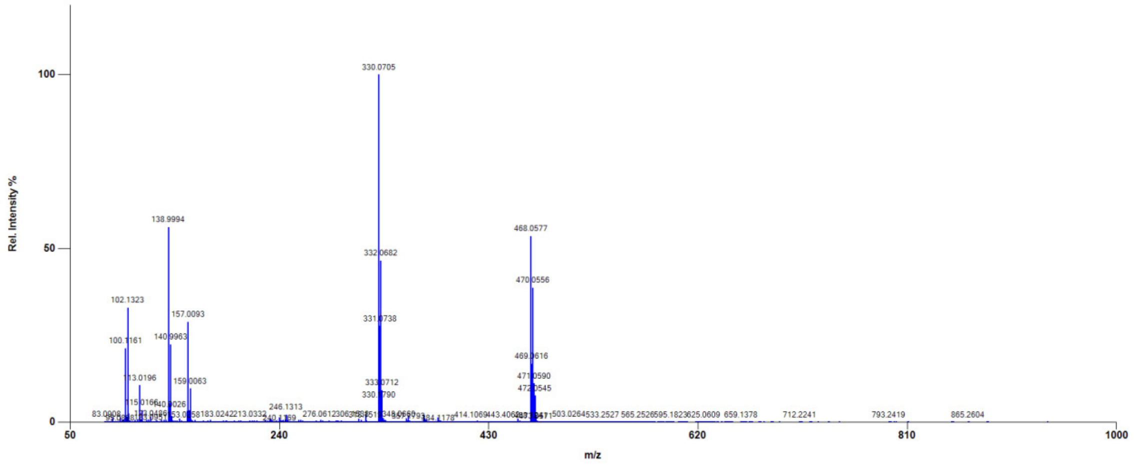

HRMS of compound 2m

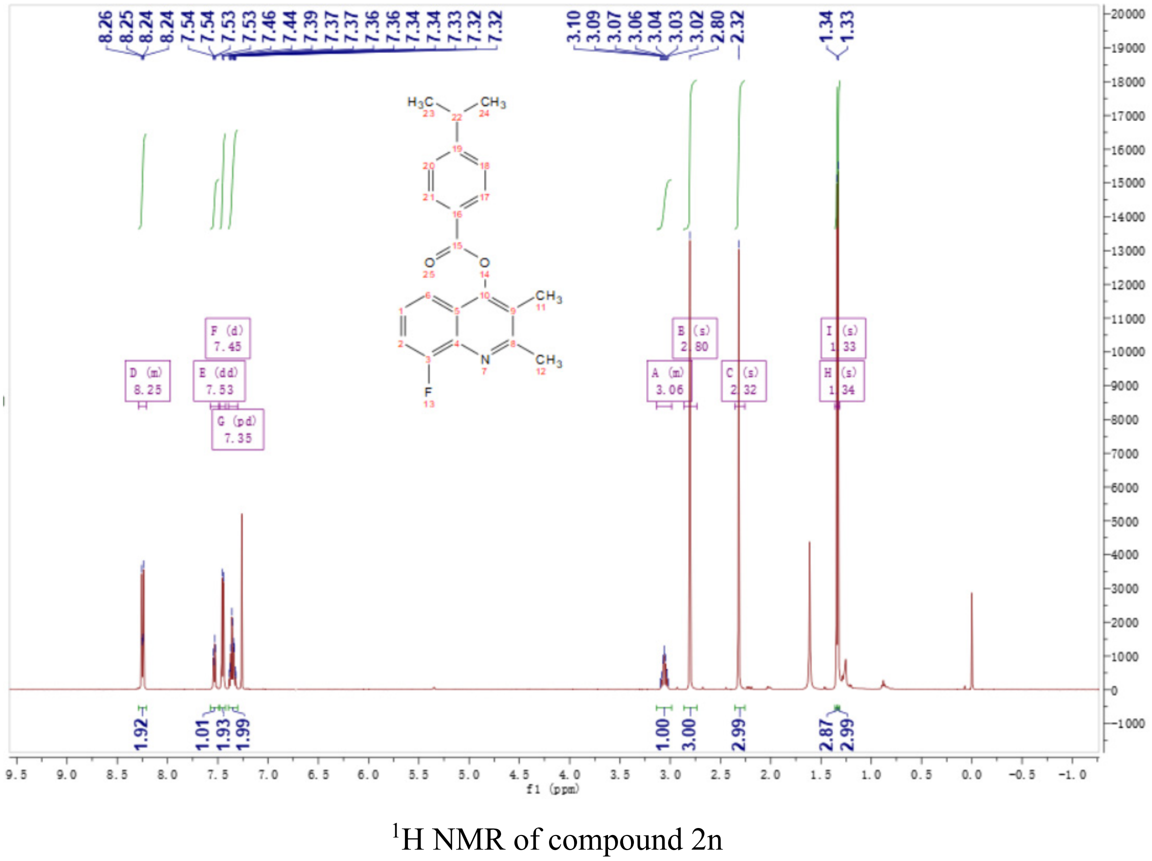

^{1}H NMR of compound 2n

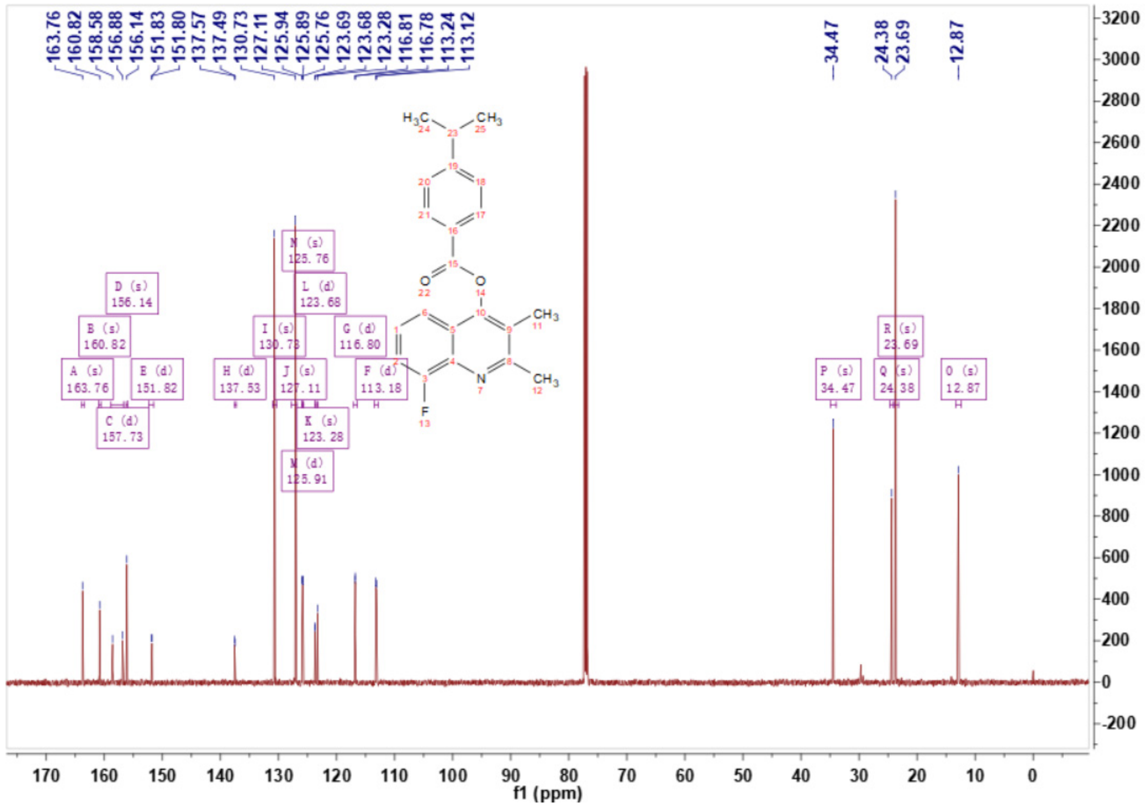

^{13}C NMR of compound 2n

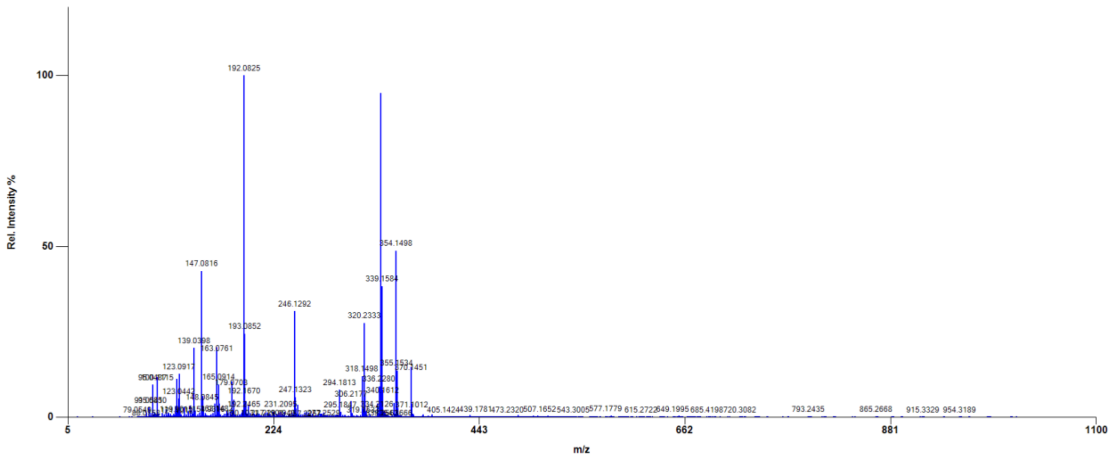

HRMS of compound 2n

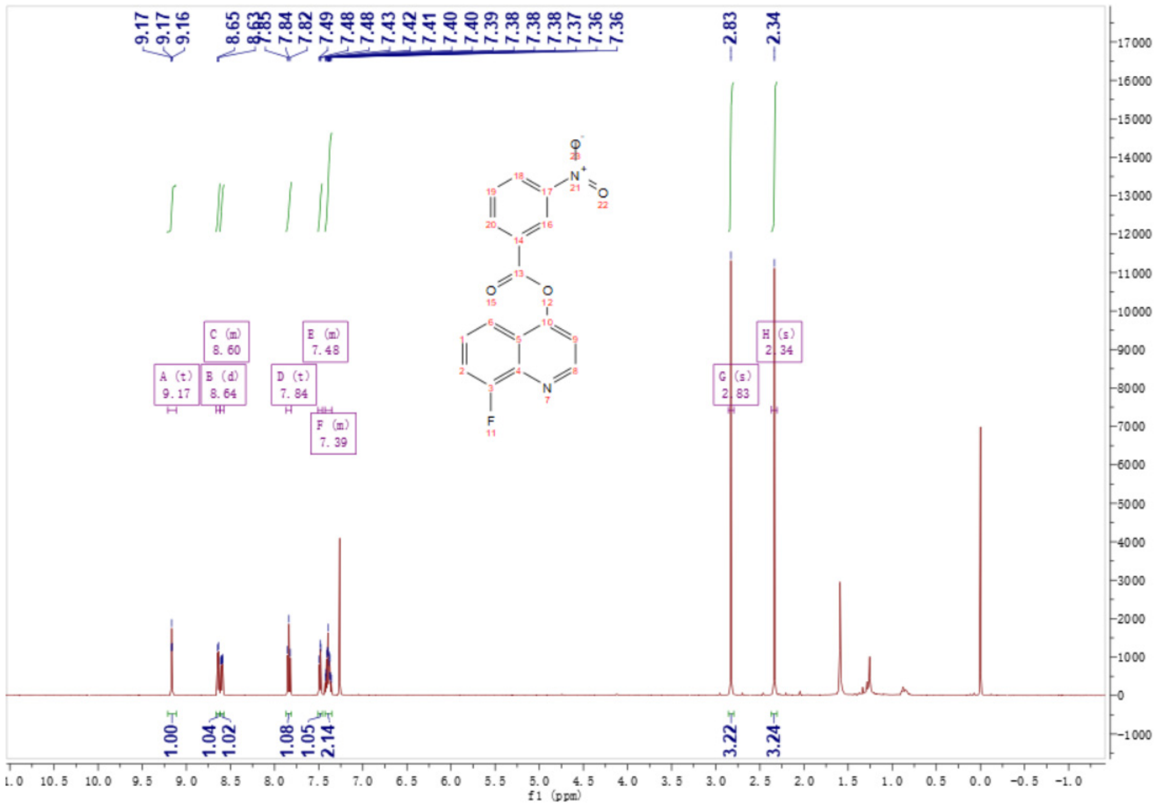

^{1}H NMR of compound 2o

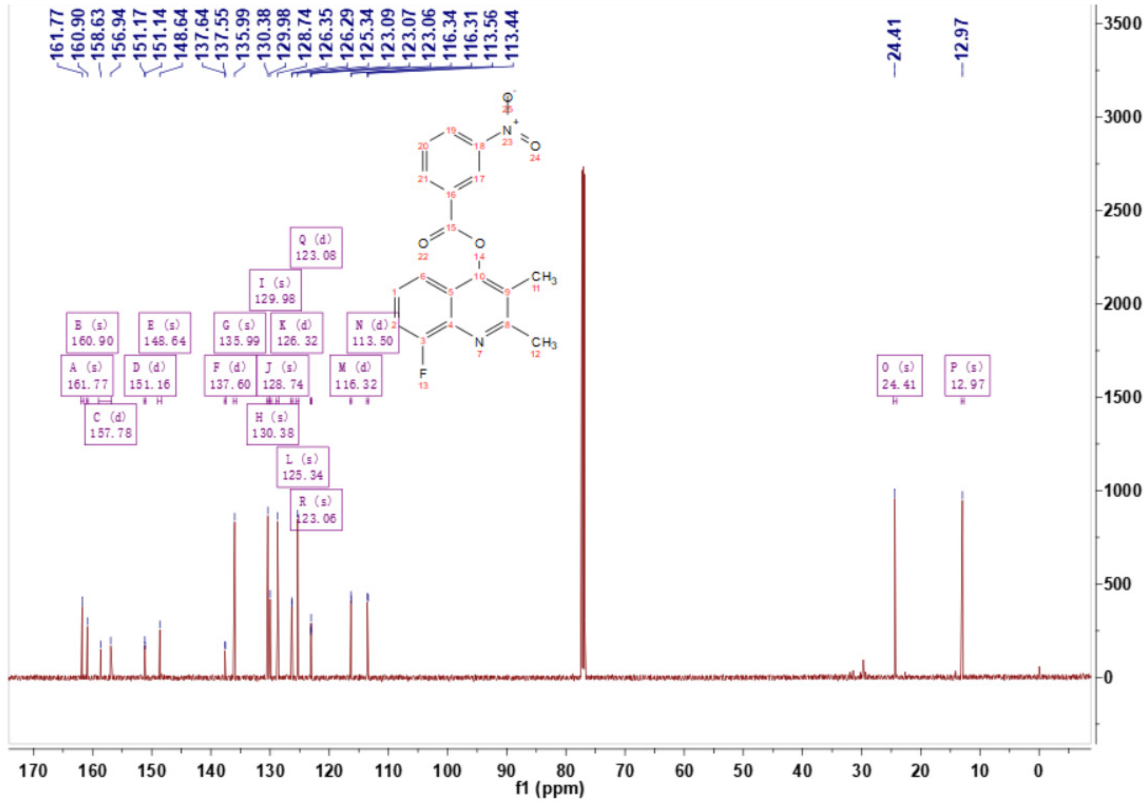

^{13}C NMR of compound 2o

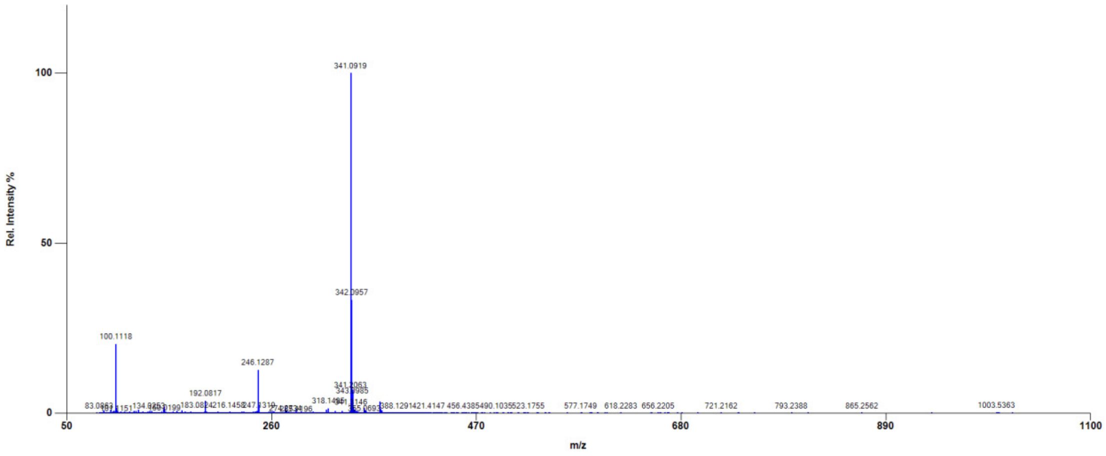

HRMS of compound 2o

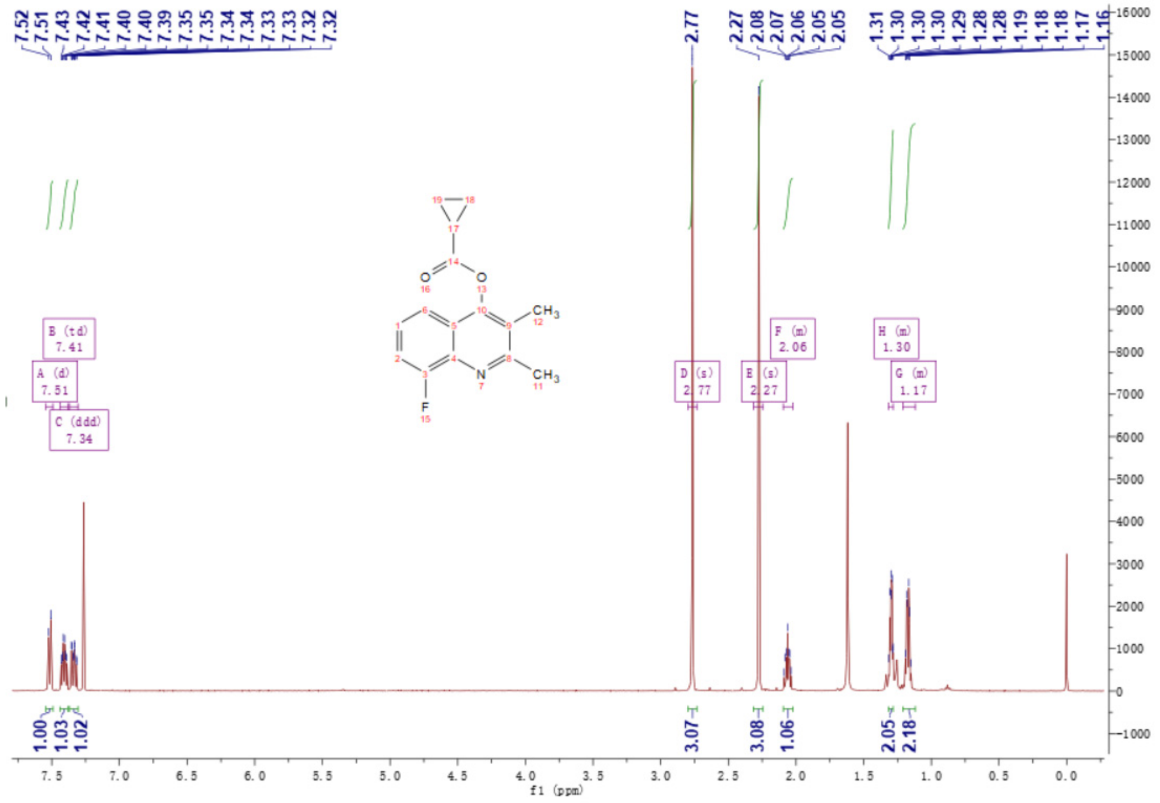

^{1}H NMR of compound 2p

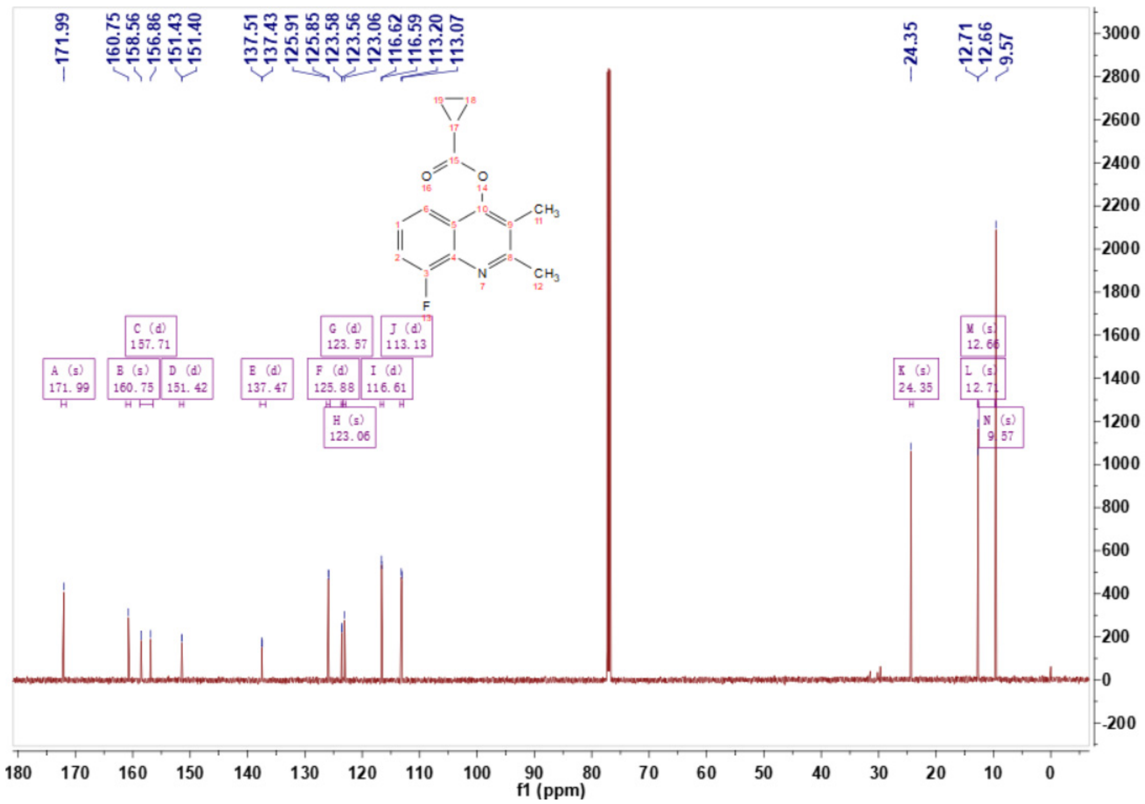

^{13}C NMR of compound 2p

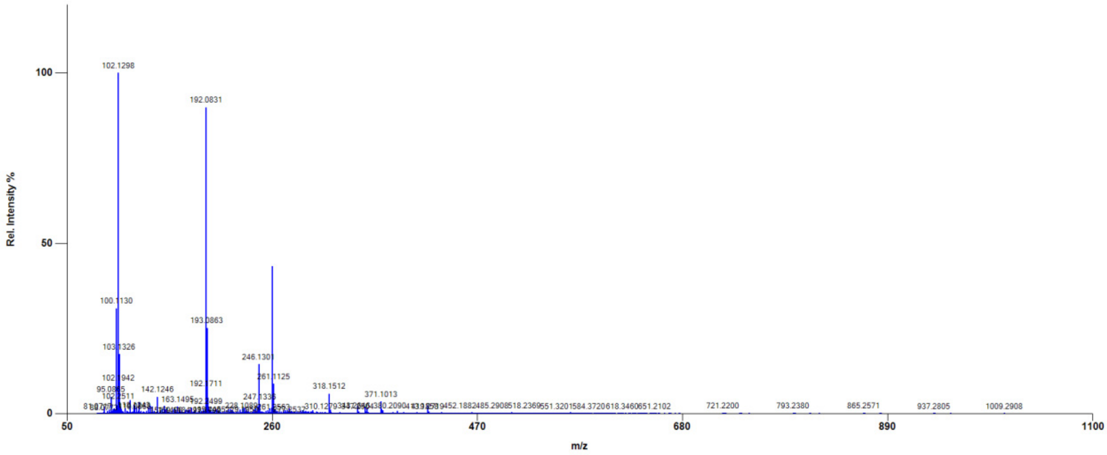

HRMS of compound 2p
